# Supplementary material for: Triphenylphosphonium Analogs of Short Peptide Related to Bactenecin 7 and Oncocin 112 as Antimicrobial Agents
Source: Pharmaceutics. 2024 Jan 22;16(1):148. doi: 10.3390/pharmaceutics16010148 (PMC10818380; doi:10.3390/pharmaceutics16010148)
Supplement: Supplementary file 1 [file pharmaceutics-16-00148-s001.zip › pharmaceutics-2730252-supplementary.pdf]

Supplementary Materials

FOR THE ARTICLE

Triphenylphosphonium Analogs of Short Peptide Related to Bacteneсin 7 and Oncocin 112 as Antimicrobial Agents

Andrey G. Tereshchenkov [1,2], Zimfira Z. Khairullina [1], Inna A. Volynkina [1], Dmitrii A. Lukianov [1], Pavel A. Nazarov [2,*], Julia A. Pavlova [1,2], Vadim N. Tashlitsky [1], Elizaveta A. Razumova [1], Daria A. Ipatova [1], Yury V. Timchenko [1], Dmitry A. Senko [3], Olga V. Efremenkova [4], Alena Paleskava [5,6], Andrey L. Konevega [5,6,7], Ilya A. Osterman [1], Igor A. Rodin [1], Petr V. Sergiev [1,2,8], Olga A. Dontsova [1,2,3], Alexey A. Bogdanov [1,2,3] and Natalia V. Sumbatyan [1,*]

[1] Department of Chemistry, Lomonosov Moscow State University, 1/3 Leninskie Gory, 119991 Moscow, Russia
[2] A.N. Belozersky Institute of Physico-Chemical Biology, Lomonosov Moscow State University, 1/40 Leninskie Gory, 119991 Moscow, Russia
[3] Shemyakin-Ovchinnikov Institute of Bioorganic Chemistry, Russian Academy of Sciences, 117997 Moscow, Russia
[4] Gause Institute of New Antibiotics, 11 B. Pirogovskaya Street, 119021 Moscow, Russia
[5] Molecular and Radiation Biophysics Division, Petersburg Nuclear Physics Institute, NRC "Kurchatov Institute", 188300 Gatchina, Russia
[6] Institute of Biomedical Systems and Biotechnology, Peter the Great St. Petersburg Polytechnic University, 195251 St. Petersburg, Russia
[7] NBICS center, NRC "Kurchatov Institute", 123182 Moscow, Russia
[8] Institute of Functional Genomics, Lomonosov Moscow State University, 119991 Moscow, Russia
* Correspondence: nazarovpa@gmail.com (P.A.N.); sumbtyan@belozersky.msu.ru (N.V.S.)

This file includes:

I. Detailed synthesis
II. Supplementary figures
III. HRMS detailed data
IV. HPLC data
V. MTT-assay data
VI. Supplementary references

I. DETAILED SYNTHESIS

Detailed Synthesis of Oncocin (Onc112)

Solid-phase synthesis of oncocin (VDKPPYLPRPRPPRrIYNr-NH_2) was carried out using custom-made automated parallel peptide synthesizer. Fmoc strategy with HATU/DIPEA activation was applied. TentaGel HL NH2 resin (loading 0.48 mmol/g) was used as solid support. Resin was functionalized by Fmoc-RAM linker. Standard protected amino acids used in this work had the following side protections:

Fmoc-Arg(Pbf)-OH, Fmoc-Asn(Trt)-OH, Fmoc-Asp(tBu)-OH, Fmoc-Lys(Boc)-OH, Fmoc-Tyr(tBu)-OH. After the synthesis, linear peptide was totally deprotected and cleaved from the polymer with TFA/DTT/H_2O/TIS (89:5:5:1) cocktail. A crude peptide was isolated by ether precipitation and subsequent purification was performed by reverse-phase HPLC with YMC Triart C18 column (150 × 30 mm). Purity (>98%) of the oncocin was confirmed by UPLC/MS analysis: *m/z* calculated for $[C_{109}H_{177}N_{37}O_{24}+H]^+$: 2390.4; found 2390.8. See also Section IV of Supplementary materials for HPLC analysis data (Figure S10.1).

Detailed Synthesis of Peptides 1 and 2

Peptidyl-polymers *Fmoc-Arg(Pbf)Arg(Pbf)IleArg(Pbf)ProArg(Pbf)ProProTyr(tBu)Leu-**P*** and *Fmoc-Arg(Pbf)Arg(Pbf)IleArg(Pbf)ProArg(Pbf)ProProArg(Pbf)Leu-**P*** were synthesized according to the standard Fmoc solid phase peptide synthesis protocol using a 2-chlorotrityl chloride resin (1.6 mmol Cl^-/g) [1] and HBTU as an activating agent. The volume of solvent was determined based on the calculation of 10 mL of solvent per 1 g of resin. The resin was prepared for synthesis by soaking it in DMF for 10–15 minutes, followed by washing with dioxane (1 × 4 min), DMF (1 × 4 min), and CH_2Cl_2 (1 × 4 min). The first amino acid addition was accomplished by adding Fmoc-Leu-OH (2 eq) and DIPEA (2 eq) in CH_2Cl_2 to the reactor and shaking for 10 minutes. An additional 3 equivalents of DIPEA were then added and mixed for an hour. At the end of the reaction, methanol was added and the mixture was shaken for 10 minutes. Then the solvent was separated by filtration, and the resin was washed successively with methylene chloride, DMF, and methanol three times each. The resin was dried in a vacuum desiccator, the loading was calculated. Further peptide chain elongation was done according to the standard protocol [1]: (1) washing resin with DMF (3 × 1 min); (2) removing of Fmoc group by 20% piperidine in DMF (2 × 15 min); (3) determination of loading using Fmoc-test [2]; (4) washing the peptidyl-resin with DMF (3 × 1 min); (5) amino acid activation: HBTU (3 eq) and DIPEA (3 eq) in DMF were mixed with amino- and side chain protected amino acid (3 eq) and stirred for 5 min; (6) condensation: the activated amino- and side chain protected amino acid was added to the peptidyl-resin and stirred for 1 h; (7) DIPEA (0.6 eq) was added after 10 min from the beginning of condensation; (8) the peptidyl-resin was filtered and washed alternately with DMF (2 × 1 min) and iPrOH (2 × 1 min); (9) completion of the condensation was monitored using Kaiser-test [3]. If the test was positive, then steps 5–9 were repeated, otherwise the next step was performed; (10) unreacted α-amino groups were blocked using a freshly prepared mixture of acetic anhydride – DIPEA – DMF (5:6:89, v/v) (1 × 5 min, 1 × 30 min); (11) the peptidyl-resin was washed with DMF (3 × 1 min). The protocol was repeated until required length of the peptide chain was reached. In the final stage, the resin was washed sequentially with DMF (1 × 5 min) and CH_2Cl_2 (1 × 5 min).

*H-ArgArgIleArgProArgProProArgLeu-OH*6TFA (**1**, Bac(1–10))*

Fmoc-Arg(Pbf)Arg(Pbf)IleArg(Pbf)ProArg(Pbf)ProProArg(Pbf)Leu-**P** was Fmoc-deprotected with 20% piperidine in DMF as described above, washed with DMF (3 × 1 min) and then treated with reagent K (TFA/phenol/water/thioanisole/mercaptoethanol, 82.5:5:5:5:2.5, 10 mL per 1 g of resin) for 4 h. Then the solution was separated by filtration, and the product was precipitated from the filtrate with diethyl ether. The target product was purified by preparative HPLC in a gradient of CH_3CN in 0.05% TFA from 5 to 30% for 15 min: τ = 12.0 min; MALDI-TOF MS: *m/z* calculated for $[C_{57}H_{105}N_{25}O_{11}+H]^+$: 1316.8; found 1316.8;

HRMS: *m/z* calculated for $[C_{57}H_{105}N_{25}O_{11}+2H]^{2+}$: 658.9286; found 658.9285. See also Sections III and IV of Supplementary materials for detailed HRMS analysis (Table S1, Figures S5.1–S5.5) and HPLC data (Figure S10.2).

*H-ArgArgIleArgProArgProProTyrLeu-OH*5TFA (**2**, Bac(1–10, R/Y))* was prepared from Fmoc-Arg(Pbf)Arg(Pbf)IleArg(Pbf)ProArg(Pbf)ProProTyr(tBu)Leu-**P** according procedure described above for compound **1**. The target product was purified by preparative HPLC in a gradient of CH_3CN in 0.05% TFA from 5 to 30% for 15 min: τ = 11.8 min; MALDI-TOF MS: m/z calculated for $[C_{60}H_{102}N_{22}O_{12}+H]^{+}$: 1323.8; found 1323.7; HRMS: m/z calculated for $[C_{60}H_{102}N_{22}O_{12}+2H]^{2+}$: 662.4097; found 662.4100. See also Sections III and IV of Supplementary materials for detailed HRMS analysis (Table S1, Figures S6.1–S6.4) and HPLC data (Figure S10.3).

Detailed Synthesis of Bac(1–10, R/Y)-C2-TPP (3)

(2-Bromoethyl)(triphenyl)phosphonium bromide ([TPP-C2-Br]Br)

1.54 g (0.0059 mol) of triphenylphosphine and 2.18 g (0.012 mol) of 1,2-dibromoethane were dissolved in methanol. The mixture was sealed in a screw-capped vessel and kept in an argon atmosphere at 85 °C for 72 h. The solvent was removed on a rotary evaporator, the resulting residue was dissolved in CH_2Cl_2 and precipitated with diethyl ether. The target product was isolated on a silica gel column eluting with a solvent system of $CHCl_3$:CH_3OH = 9:1. Yield: 2.54 g (96%); TLC: R_f ($CHCl_3$:CH_3OH, 9:1) 0.61; LC-MS *m/z* calculated for $[C_{20}H_{19}BrP]^{+}$: 369.04; found 369.30; τ (UPLC) = 1.53 min; ^{1}H NMR ($CDCl_3$): 7.87–7.75 (m, 12H, *ortho*-H^{Ph}, *meta*-H^{Ph}), 7.70–7.63 (m, 3H, *para*-H^{Ph}), 4.57 (dt, 2H, *J* = 11.9, 6.5 Hz, CH_2-P^+Ph_3), 3.74 (dt, 2H, *J* = 19.3, 6.5 Hz, CH_2-Br); ^{13}C NMR ($CDCl_3$): 135.41 (d, 3C, $J_{C,P}$ = 3.2 Hz, *para*-C^{Ph}), 133.96 (d, 6C, $J_{C,P}$ = 10.3 Hz, *ortho*-C^{Ph}), 130.58 (d, 6C, $J_{C,P}$ = 12.9 Hz, *meta*-C^{Ph}), 117.35 (d, 3C, $J_{C,P}$ = 86.3 Hz, *ipso*-C^{Ph}), 27.08 (d, $J_{C,P}$ = 52.0 Hz, -CH_2-P^+Ph_3), 22.90 (d, $J_{C,P}$ = 5.2 Hz, CH_2-Br); ^{31}P NMR ($CDCl_3$): 23.34 (s).

(2-Aminoethyl)(triphenyl)phosphonium bromide ([TPP-C2-NH₂]Br)

0.5 g (0.0011 mol) of [TPP-C2-Br]Br was dissolved in 10 mL of 7 N ammonia solution in methanol and the mixture was kept for 4 h at 85 °C. Then the mixture was cooled, and volatile components were removed on a rotary evaporator. The target product was isolated on a silica gel column eluting with a solvent system of $CHCl_3$:CH_3OH = 6:1. Yield: 0.246 g (58%); TLC: R_f ($CHCl_3$:CH_3OH, 6:1) 0.76; LC-MS *m/z* calculated for $[C_{20}H_{21}NP]^{+}$: 306.14; found 306.31; τ (UPLC) = 0.76 min; ^{1}H NMR (DMSO-d_6): 7.97–7.90 (m, 3H, *para*-H^{Ph}), 7.89–7.75 (m, 12H, *ortho*-H^{Ph}, *meta*-H^{Ph}), 4.07–3.95 (m, 2H, -CH_2-P^+Ph_3), 3.14–3.04 (m, 2H, NH_2-$C\underline{H}_2$-); ^{13}C NMR (DMSO-d_6): 135.41 (d, 3C, $J_{C,P}$ = 3.1 Hz, *para*-C^{Ph}), 133.73 (d, 6C, $J_{C,P}$ = 10.7 Hz, *ortho*-C^{Ph}), 130.46 (d, 6C, $J_{C,P}$ = 12.8 Hz, *meta*-C^{Ph}), 117.26 (d, 3C, $J_{C,P}$ = 87.1 Hz, *ipso*-C^{Ph}), 33.35 (d, $J_{C,P}$ = 2.8 Hz, NH_2-CH_2-), 19.62 (d, $J_{C,P}$ = 54.7 Hz, -CH_2-P^+Ph_3); ^{31}P NMR (DMSO-d_6): 21.33 (s).

Fmoc-Arg(Pbf)Arg(Pbf)IleArg(Pbf)ProArg(Pbf)ProProTyr(tBu)Leu-C2-TPP

Peptidyl-polymer Fmoc-Arg(Pbf)Arg(Pbf)IleArg(Pbf)ProArg(Pbf)ProProTyr(tBu)Leu-**P** was treated with a 50% solution of HFIP in CH_2Cl_2 for 1h. Volatile components were removed using a rotary

evaporator. Obtained peptide with protected groups Fmoc-Arg(Pbf)Arg(Pbf)IleArg(Pbf)ProArg(Pbf)ProProTyr(tBu)Leu-COOH (**6**) was isolated on a silica gel column eluting with a solvent system of $CHCl_3$:CH_3OH = 4:1 (R_f 0.33). Next, compound **6** (84 mg, 0.032 mmol), DIPEA (17 µl, 0.097 mmol), and HBTU (16 mg, 0.042 mmol) were dissolved in CH_2Cl_2 and kept for 5 min. Then [TPP-C2-NH_2]Br (19 mg, 0.048 mmol) was added to the solution. The obtained mixture was stirred for 1.5 h. Then the solvent was removed on a rotary evaporator, the resulting residue was dissolved in ethyl acetate and washed sequentially with water, 0.1 N H_2SO_4, saturated $NaHCO_3$, and saturated NaCl, dried over anhydrous sodium sulfate, then the solvent was removed on a rotary evaporator. The target product was isolated on a silica gel column eluting with a solvent system of $CHCl_3$:CH_3OH = 9:1. Yield: 76 mg (81%); TLC: R_f ($CHCl_3$:CH_3OH, 9:1) 0.29; MALDI-TOF MS: *m/z* calculated for $[C_{151}H_{203}N_{23}O_{25}PS_4]^+$: 2897.4; found 2897.6.

*H-ArgArgIleArgProArgProProTyrLeu-C2-TPP*6TFA (**3**, Bac(1–10, R/Y)-C2-TPP)*

Fmoc-Arg(Pbf)Arg(Pbf)IleArg(Pbf)ProArg(Pbf)ProProTyr(tBu)Leu-C2-TPP (76 mg, 0.026 mmol) was treated with reagent K by the procedure described for compound **1**. After precipitation with diethyl ether, the residue was mixed with 1.5 mL of 20% piperidine in DMF and stirred for 1.5 h. The product was precipitated with diethyl ether, and the solid precipitate was washed three times with ether. The target product was isolated by preparative HPLC in a gradient of CH_3CN in 0.05% TFA from 20 to 40% for 18 min: τ = 14.5 min. Yield: 20 mg (33%); MALDI-TOF MS: *m/z* calculated for $[C_{80}H_{121}N_{23}O_{11}P]^+$: 1610.9; found 1610.9; HRMS: *m/z* calculated for $[C_{80}H_{121}N_{23}O_{11}P+H]^{2+}$: 805.9710; found 805.9708. See also Sections III and IV of Supplementary materials for detailed HRMS analysis (Table S1, Figures S7.1–S7.5) and HPLC data (Figure S10.4).

Detailed Synthesis of Bac(1–10, R/Y)-C10-TPP (4)

(10-Bromodecyl)(triphenyl)phosphonium bromide ([TPP-C10-Br]Br)

3 g of triphenylphosphine (0.011 mol) and 4.5 g of 1,10-dibromodecane (0.015 mol) were dissolved in ethanol. The obtained mixture was sealed in a screw-capped vessel and kept in an argon atmosphere at 85 °C for 72 h. The solvent was removed on a rotary evaporator, the resulting residue was dissolved in CH_2Cl_2 and precipitated with diethyl ether. The target product was isolated on a silica gel column eluting with a solvent system of $CHCl_3$:CH_3OH = 9:1. Yield: 5.32 g (83%); TLC: R_f ($CHCl_3$:CH_3OH, 9:1) 0.33; LC-MS *m/z* calculated for $[C_{28}H_{35}BrP]^+$: 481.2; found 482.7; ^1H NMR ($CDCl_3$): 7.78–7.64 (m, 15H, -Ph), 3.30 (dt, 2H, *J* = 15.2, 5.9, 4.9 Hz, Ph_3P^+-CH_2-), 2.55 (t, 2H, *J* = 7.3 Hz, -CH_2-Br), 1.74–1.53 (m, 6H, Ph_3P^+-CH_2-C$\underline{H}_2$-C$\underline{H}_2$-, -C$\underline{H}_2$-CH_2-Br), 1.36–1.23 (m, 10H, -CH_2-C$\underline{H}_2$-CH_2-); ^{13}C NMR ($CDCl_3$): 135.10 (d, 3C, $J_{C,P}$ = 3.0 Hz, *para*-C^{Ph}), 133.60 (d, 6C, $J_{C,P}$ = 10.0 Hz, *ortho*-C^{Ph}), 130.58 (d, 6C, $J_{C,P}$ = 12.5 Hz, *meta*-C^{Ph}), 117.56 (d, 3C, $J_{C,P}$ = 86.0 Hz, *ipso*-C^{Ph}), 34.19 (CH_2-Br), 32.24–22.52 (9C, -CH_2-).

(10-Aminodecyl)(triphenyl)phosphonium bromide ([TPP-C10-NH_2]Br) was prepared as described above for [TPP-C2-Br]Br from 1 g of [TPP-C10-Br]Br (0.0018 mol) and 20 mL of 7 N ammonia solution in methanol. The product was isolated on a silica gel column eluting with a solvent system of $CHCl_3$:CH_3OH

= 4:1. Yield: 481 mg (54%); TLC: R_f ($CHCl_3$:CH_3OH, 4:1) 0.36; LC-MS *m/z* calculated for $[C_{28}H_{37}NP]^+$: 418.27; found 418.37; ^{1}H NMR ($CDCl_3$): 7.83–7.76 (m, 15H, -Ph), 3.73 (br s, 2H, -N$\underline{H}_2$), 3.00 (dt, 2H, J = 15.2, 5.9, 4.9 Hz, Ph_3P^+-CH_2), 2.26 (t, 2H, J = 7.3 Hz, -C$\underline{H}_2$-NH_2), 1.89–1.63 (m, 6H, Ph_3P^+-CH_2-C$\underline{H}_2$-C$\underline{H}_2$-, -C$\underline{H}_2$-CH_2-NH_2), 1.31–1.19 (m, 10H, -CH_2-C$\underline{H}_2$-CH_2-); ^{13}C NMR ($CDCl_3$): 135.11 (d, 3C, $J_{C,P}$ = 3.0 Hz, *para*-C^{Ph}), 133.73 (d, 6C, $J_{C,P}$ = 10.0 Hz, *ortho*-C^{Ph}), 130.48 (d, 6C, $J_{C,P}$ = 12.5 Hz, *meta*-C^{Ph}), 117.58 (d, 3C, $J_{C,P}$ = 86.0 Hz, *ipso*-C^{Ph}), 40.05 (CH_2-NH_2), 32.33–22.43 (9C, -CH_2-).

*H-ArgArgIleArgProArgProProTyrLeu-C10-TPP*6TFA (**4**, Bac(1–10, R/Y)-C10-TPP)*

Fmoc-Arg(Pbf)Arg(Pbf)IleArg(Pbf)ProArg(Pbf)ProProTyr(tBu)Leu-C10-TPP was obtained by the procedure described for Fmoc-Arg(Pbf)Arg(Pbf)IleArg(Pbf)ProArg(Pbf)ProProTyr(tBu)Leu-C2-TPP starting from compound **6** (100 mg, 0.038 mmol), DIPEA (14 µl, 0.076 mmol), HBTU (21.8 mg, 0.057 mmol), and [TPP-C10-NH_2]Br (21 mg, 0.042 mmol). The target product was isolated on a silica gel column eluting with a solvent system of $CHCl_3$:CH_3OH = 9:1. Yield: 45 mg (39%). TLC: R_f ($CHCl_3$:CH_3OH, 9:1) 0.42, R_f ($CHCl_3$:CH_3OH, 6:1) 0.56. Bac(1–10, R/Y)-C10-TPP (**4**) was obtained from Fmoc-Arg(Pbf)Arg(Pbf)IleArg(Pbf)ProArg(Pbf)ProProTyr(tBu)Leu-C10-TPP (20 mg, 0.0066 mmol) by the procedure described for compound **1**. The target product was isolated by preparative HPLC in a gradient of CH_3CN in 0.05% TFA from 20 to 40% for 18 min: τ = 16.7 min. Yield: 8.5 mg (53%); MALDI-TOF MS: *m/z* calculated for $[C_{88}H_{137}N_{23}O_{11}P]^+$: 1723.1; found 1723.0; HRMS: *m/z* calculated for $[C_{88}H_{137}N_{23}O_{11}P+2H]^{3+}$: 575.0249; found 575.0249. See also Sections III and IV of Supplementary materials for detailed HRMS analysis (Table S1, Figures S8.1–S8.4) and HPLC data (Figure S10.5).

Detailed Synthesis of TPP-C10-Bac(1–10, R/Y) (5)

(10-Carboxydecyl)triphenylphosphonium bromide ([TPP-C10-COOH]Br)

1.57 g (6 mmol) of triphenylphosphine and 1.32 g (5 mmol) of 11-bromoundecanoic acid were dissolved in 4 mL of benzene and the mixture was kept for 72 hours at 85˚C. Then benzene was evaporated on a rotary evaporator. The obtained residue was dissolved in 4 mL of methylene chloride following to addition of fivefold excess of diethyl ether and left in the refrigerator to crystallize. The precipitate was separated by filtration, washed with ether and dried over $CaCl_2$. Yield: 2.13 g (81%); TLC: R_f ($CHCl_3$:MeOH, 9:1) 0.33; LC-MS *m/z* calculated for $C_{29}H_{36}O_2P$ $(M)^+$: 447.24, found 447.82; ^{1}H NMR ($CDCl_3$): 7.85–7.72 (m, 15H, -Ph), 3.40 (dt, 2H, J = 15.2, 5.9, 4.9 Hz, Ph_3P^+-CH_2-), 2.35 (t, 2H, J = 7.3 Hz, -C$\underline{H}_2$-COOH), 1.72–1.55 (m, 6H, Ph_3P^+-CH_2-C$\underline{H}_2$-C$\underline{H}_2$-, -C$\underline{H}_2$-CH_2-COOH), 1.36–1.23 (m, 10H, -CH_2-C$\underline{H}_2$-CH_2-); ^{13}C NMR ($CDCl_3$): 177.53 (COOH), 135.14 (d, 3C, $J_{C,P}$ = 3.0 Hz, *para*-C^{Ph}), 133.61 (d, 6C, $J_{C,P}$ = 10.0 Hz, *ortho*-C^{Ph}), 130.58 (d, 6C, $J_{C,P}$ = 12.5 Hz, *meta*-C^{Ph}), 118.22 (d, 3C, $J_{C,P}$ = 86.0 Hz, *ipso*-C^{Ph}), 34.45 (-$\underline{C}H_2$-COOH), 30.38–22.42 (9C, -CH_2-); ^{31}P NMR ($CDCl_3$) 23.80 (s).

*TPP-C10-C(O)NH-ArgArgIleArgProArgProProTyrLeu-OH*5TFA (**5**, TPP-C10-Bac(1–10, R/Y))*

150 mg of peptidyl-polymer Fmoc-Arg(Pbf)Arg(Pbf)IleArg(Pbf)ProArg(Pbf)ProProTyr(tBu)Leu-**P** was treated with 20% solution of piperidine in DMF for 15 min. Next, the solvent was removed by filtration and the resin was washed with DMF several times. [TPP-C10-COOH]Br (211 mg, 0.40 mmol), HBTU (152

mg, 0.40 mmol), DIPEA (70 μL, 0.40 mmol) were added to peptidyl-polymer in DMF and stirred for 12 h. Then the peptidyl polymer was washed with DMF and CH_2Cl_2 and treated with 3 mL of reagent K for 4 h. The product was precipitated with diethyl ether, and the solid precipitate was washed three times with diethyl ether. Yield: 82 mg (63%). The target product was isolated by preparative HPLC in a gradient of CH_3CN in 0.05% TFA from 20 to 40% for 18 min: τ = 11.5 min. MALDI-TOF MS: m/z calculated for $[C_{89}H_{136}N_{22}O_{13}P]^+$: 1752.0; found 1751.9; HRMS: *m/z* calculated for $[C_{89}H_{136}N_{22}O_{13}P+H]^{2+}$: 877.0248; found 877.0250. See also Sections III and IV of Supplementary materials for detailed HRMS analysis (Table S1, Figures S9.1–S9.2) and HPLC data (Figure S10.6).

Detailed Synthesis of BODIPY-ArgArgIleArgProArgProProArgLeu-OH*5TFA (BODIPY-Bac(1–10))

4 mg (0.0020 mmol) of Bac(1–10)*6TFA was dissolved in 200 μL of 0.2 M $NaHCO_3$ and 0.7 mg (0.0018 mmol) of succinimide ester BODIPY-FL-C3 in 40 μL of DMF was added. The resulting mixture was stirred for 2 h at room temperature. The obtained mixture was diluted 10 times with water and the product was purified by preparative HPLC in a gradient of CH_3CN in 0.05% TFA from 5 to 40% for 20 min: τ = 17.3 min. Yield: 1.2 mg (31%); fluorescence (MeOH): λ_{ex} = 505 nm, λ_{em} = 510 nm; LC-MS *m/z* calculated for $[C_{71}H_{118}BF_2N_{27}O_{12}+2H]^{2+}$: 795.98; found 794.82; τ (UPLC) = 0.89 min; MALDI-TOF MS *m/z* calculated for $[C_{71}H_{118}BF_2N_{27}O_{12}+H]^+$: 1591.0; found 1591.0.

II. SUPPLEMENTARY FIGURES

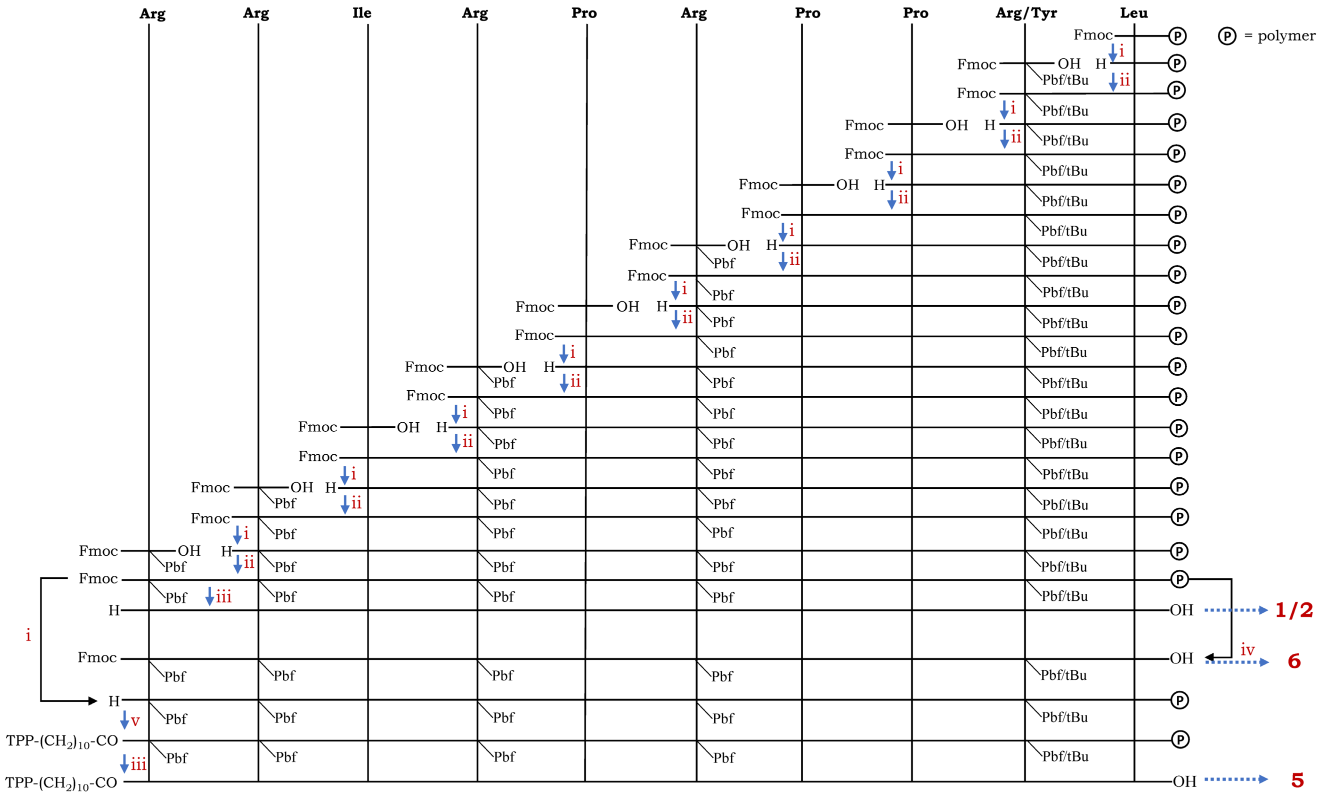

Figure S1. Scheme of the chemical synthesis of short peptide from Bac 7 (Bac(1–10), **1**), its R[9]/Y-analog (Bac(1–10, R/Y), **2**), their triphenylphosphonium analog TPP-C10-Bac(1–10, R/Y), **5**, and protected peptide **6**: i – 20% Pip/DMF, ii – HBTU/DIPEA/DMF, iii – reagent K, iv – HFIP/CH_2Cl_2, v – TPP-$(CH_2)_{10}$-COOH/HBTU/DIPEA/DMF. Symbol Ⓟ stands for polymer support.

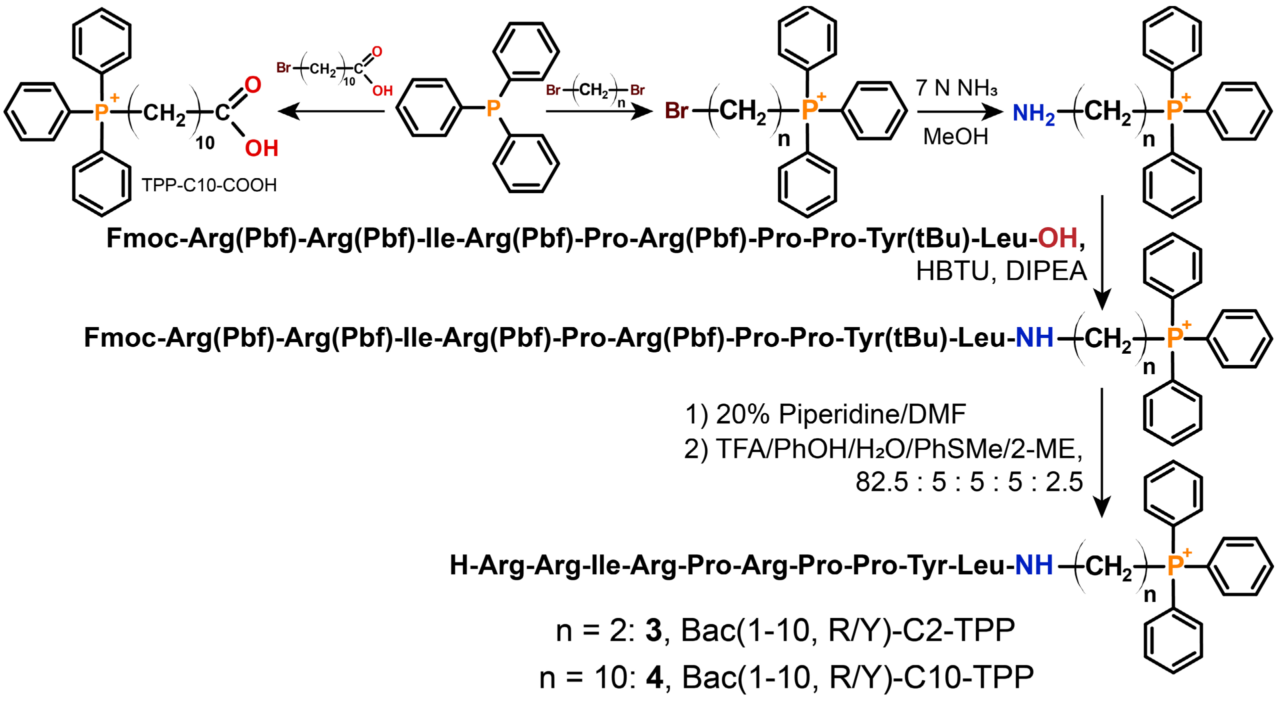

Figure S2. Scheme of the chemical synthesis of triphenylphosphonium analogs of short peptide from Bac7: (Bac(1–10, R/Y)-C2-TPP, **3**) and (Bac(1–10, R/Y)-C10-TPP, **4**).

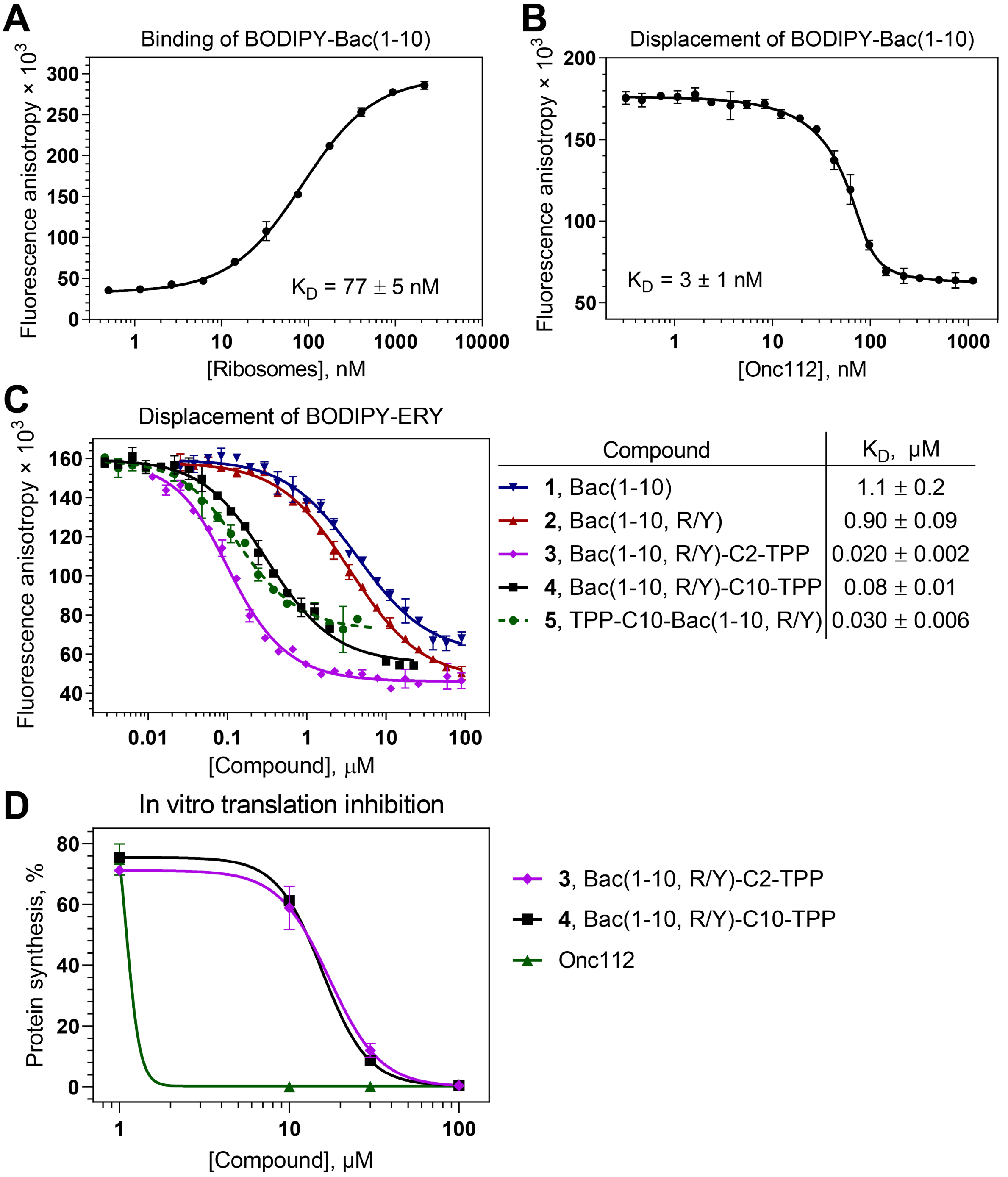

Figure S3. Binding affinity to bacterial ribosomes and in vitro inhibition of protein synthesis of triphenylphosphonium analogs of short peptide from Bac7. Average values of apparent dissociation constants (K_D) with CI ($\alpha = 0.05$) are shown. All experiments were performed at least three times, error bars are SD. (**A**) Binding of BODIPY-Bac(1–10) to *E. coli* 70S ribosomes measured by fluorescence anisotropy. (**B**) A competitive binding assay to test the affinity of Onc112 to *E. coli* 70S ribosomes measured by displacement of BODIPY-Bac(1–10). (**C**) A competitive binding assay to test the affinity of peptides **1** and **2** and their TPP analogs **3**–**5** to *E. coli* 70S ribosomes measured by displacement of BODIPY-ERY. (**D**) Inhibition of protein synthesis by increasing concentrations of Onc112, compound **3** (Bac(1–10, R/Y)-C2-TPP), and **4** (Bac(1–10, R/Y)-C10-TPP) in the in vitro cell-free *E. coli* S30 extract translation system. The relative enzymatic activity of in vitro synthesized firefly luciferase is shown. Error-bars represent the SD values.

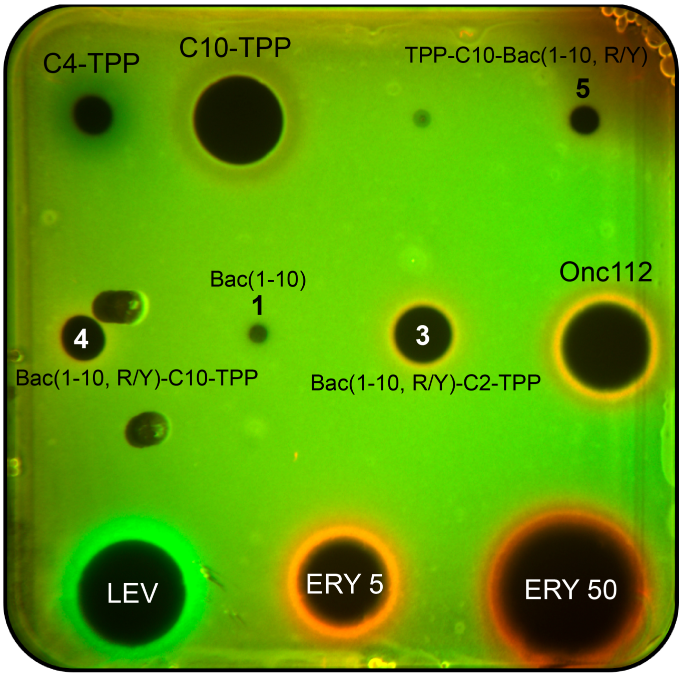

Figure S4. Testing of antibacterial activity of triphenylphosphonium derivatives of decapeptide related to Bac7 and Onc112 using *E. coli (ΔtolC)* pDualrep2 strain. Onc112, erythromycin at concentrations of 5 mg/mL (ERY 5) and 50 mg/mL (ERY 50), levofloxacin (LEV), C10-TPP, and C4-TPP are used as the controls. The induction of the red fluorescent protein expression (green halo around the inhibition zone, pseudocolor) is triggered by DNA damage, while the induction of Katushka2S protein (red halo, pseudocolor) occurs in response to ribosome stalling. Unlabeled spots correspond to substances not studied in this work.

III. HRMS DETAILED DATA

Table S1. HRMS data of triphenylphosphonium analogs of short peptide from Bac7 in positive ion mode. Calculated/measured *m*/*z* values are presented.

Compound	$[M+H]^{2+}/2$ or $*[M+2H]^{2+}/2$ calcd./meas.	$[M+2H]^{3+}/3$ or $*[M+3H]^{3+}/3$ calcd./meas.	$[M+3H]^{4+}/4$ or $*[M+4H]^{4+}/4$ calcd./meas.	$[M+4H]^{5+}/5$ or $*[M+5H]^{5+}/5$ calcd./meas.
**1**, Bac(1–10)	*658.9286/658.9285	*439.6215/439.6215	*329.9679/329.9681	*264.1758/264.1758
**2**, Bac(1–10, R/Y)	*662.4097/662.4100	*441.9422/441.9423	*331.7085/331.7084	-
**3**, Bac(1–10, R/Y)-C2-TPP	805.9710/805.9708	537.6498/537.6496	403.4892/403.4892	322.9928/322.9931
**4**, Bac(1–10, R/Y)-C10-TPP	-	575.0249/575.0249	431.5205/431.5209	345.4178/345.4180
**5**, TPP-C10-Bac(1–10, R/Y)	877.0248/877.0250	585.0189/585.0185	439.0160/439.0159	351.4143/351.4144

HRMS data for Bac(1–10) (1)

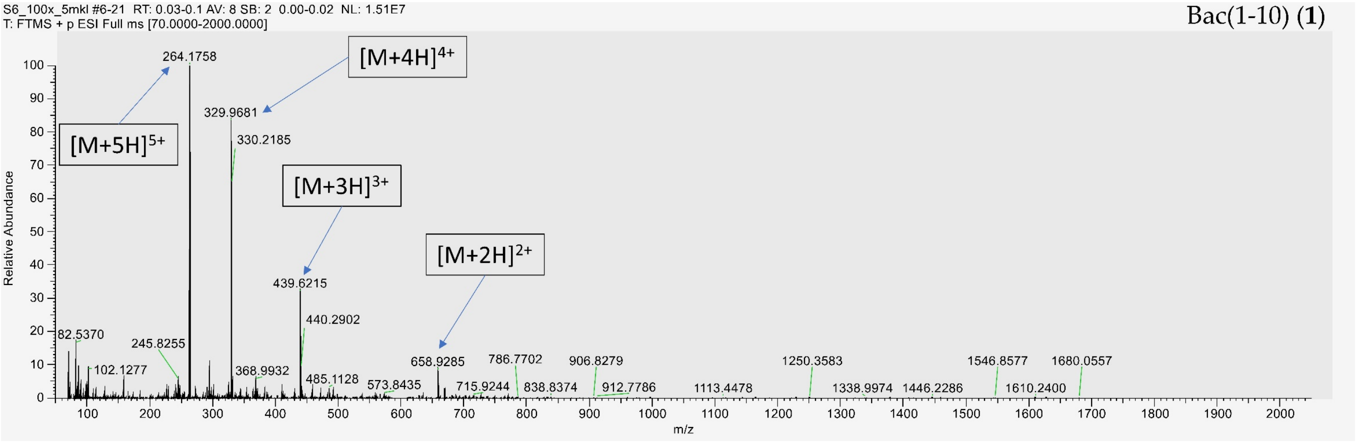

Figure S5.1. The HRMS spectrum of compound **1** in positive ion mode.

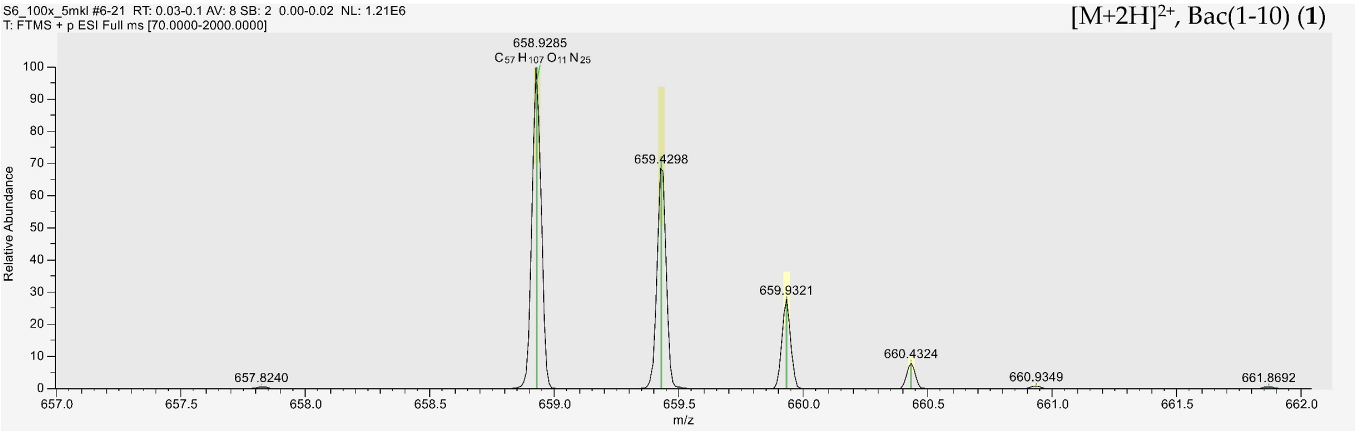

Peak Mass	Display Formula	Delta [ppm]	Theo. mass	Pattern Cov. [%]
658.9285	$C_{57}H_{107}O_{11}N_{25}$	-0.08	658.9286	99.28

Figure S5.2. The HRMS data for $[M+2H]^{2+}$ ion of $[C_{57}H_{105}N_{25}O_{11}]$ (compound **1**).

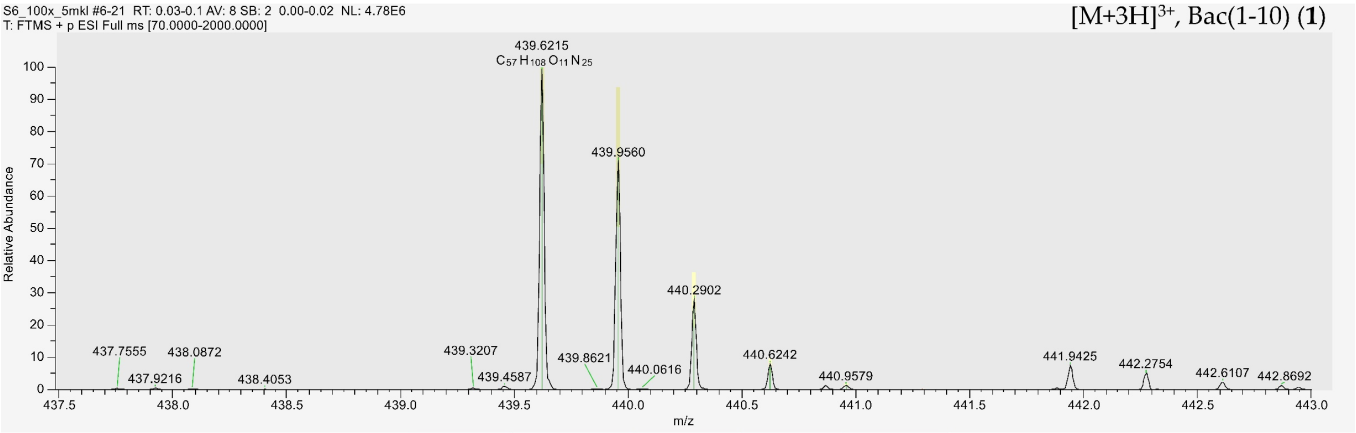

Peak Mass	Display Formula	Delta [ppm]	Theo. mass	Pattern Cov. [%]
439.6215	$C_{57}H_{108}O_{11}N_{25}$	0.12	439.6215	99.88

Figure S5.3. The HRMS data for $[M+3H]^{3+}$ ion of $[C_{57}H_{105}N_{25}O_{11}]$ (compound **1**).

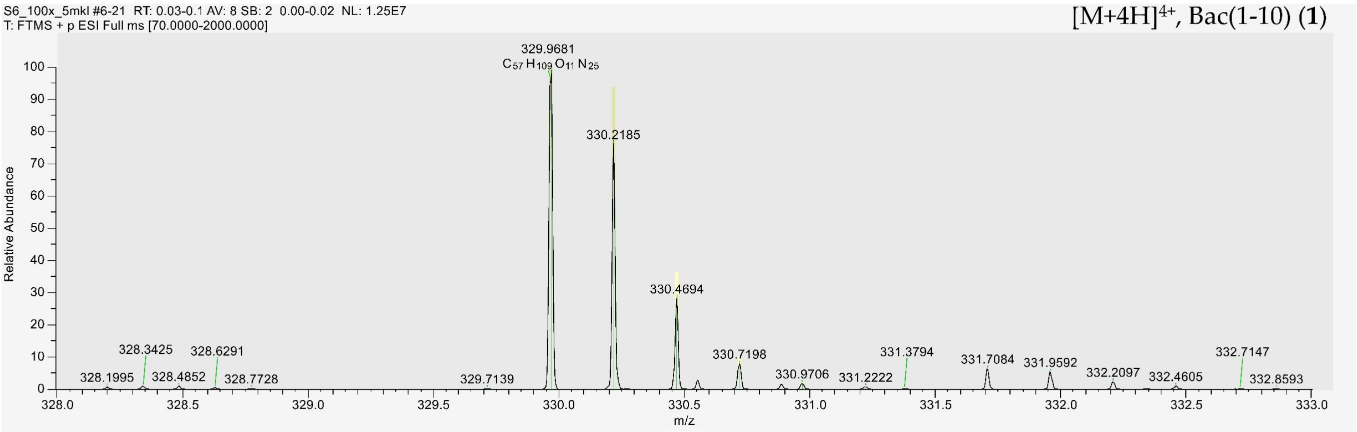

Peak Mass	Display Formula	Delta [ppm]	Theo. mass	Pattern Cov. [%]
329.9681	$C_{57}H_{109}O_{11}N_{25}$	0.44	329.9679	99.88

Figure S5.4. The HRMS data for $[M+4H]^{4+}$ ion of $[C_{57}H_{105}N_{25}O_{11}]$ (compound **1**).

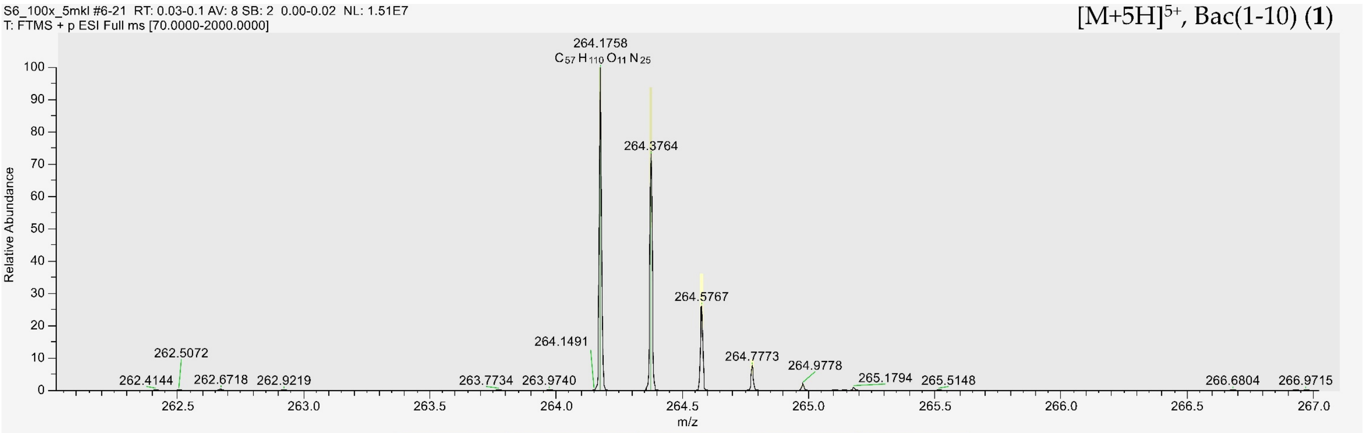

Peak Mass	Display Formula	Delta [ppm]	Theo. mass	Pattern Cov. [%]
264.1758	$C_{57}H_{110}O_{11}N_{25}$	-0.08	264.1758	99.88

Figure S5.5. The HRMS data for $[M+5H]^{5+}$ ion of $[C_{57}H_{105}N_{25}O_{11}]$ (compound **1**).

HRMS data for Bac(1–10, R/Y) (2)

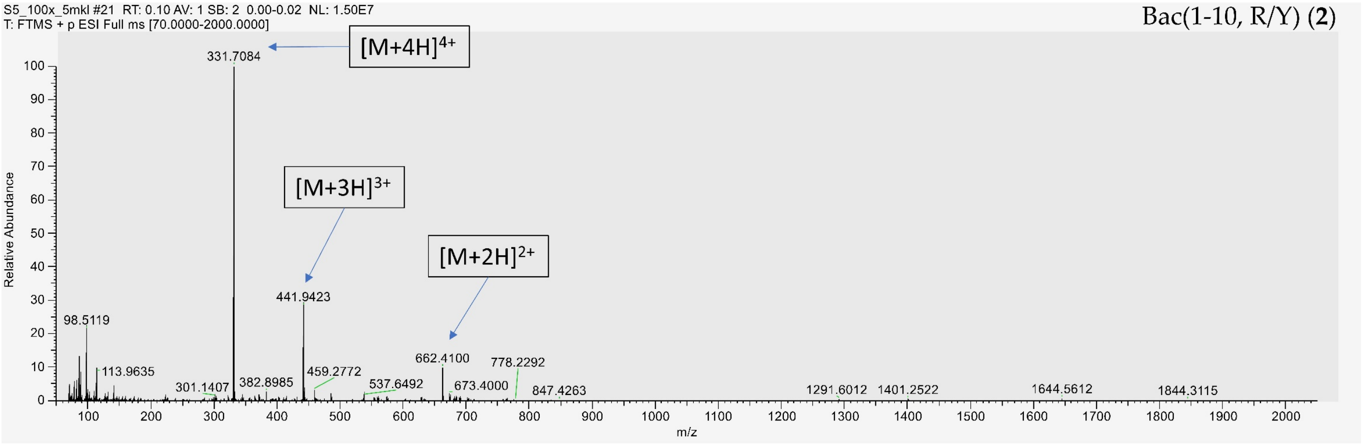

Figure S6.1. The HRMS spectrum of compound **2** in positive ion mode.

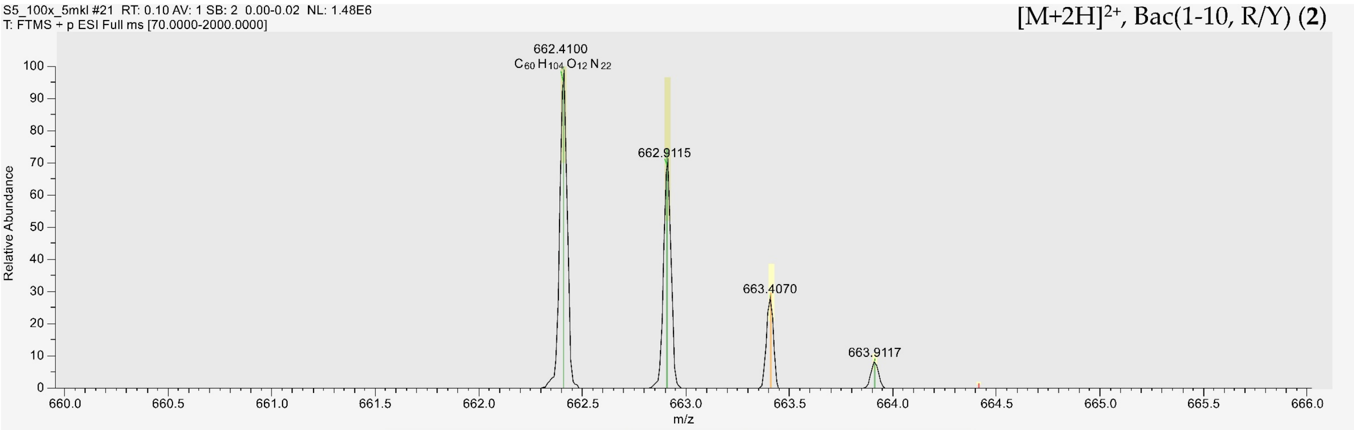

Peak Mass	Display Formula	Delta [ppm]	Theo. mass	Pattern Cov. [%]
662.41	$C_{60}H_{104}O_{12}N_{22}$	0.54	662.4097	85.29

Figure S6.2. The HRMS data for $[M+2H]^{2+}$ ion of [$C_{60}H_{102}N_{22}O_{12}$] (compound **2**).

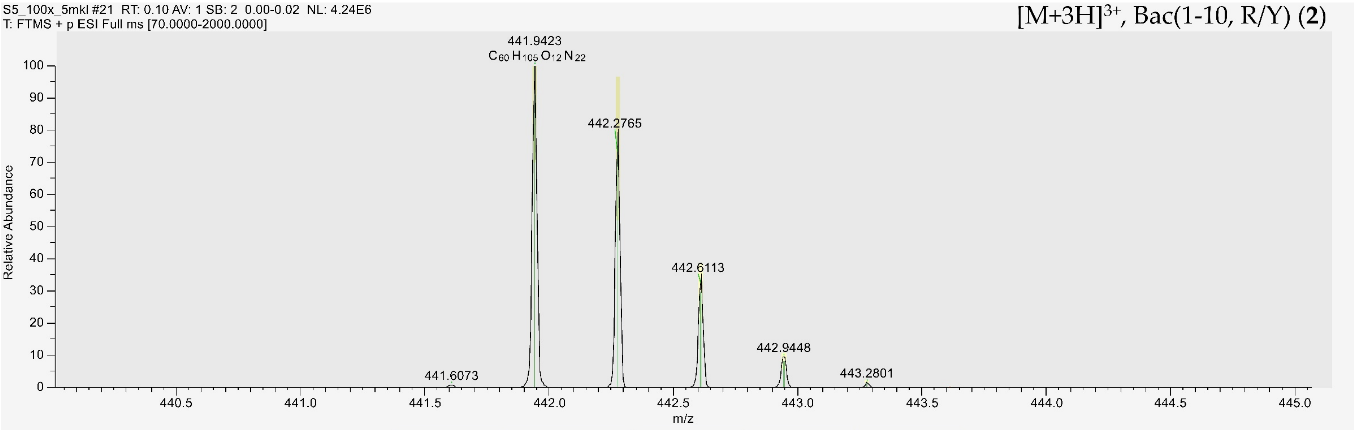

Peak Mass	Display Formula	Delta [ppm]	Theo. mass	Pattern Cov. [%]
441.9423	$C_{60}H_{105}O_{12}N_{22}$	0.34	441.9422	99.86

Figure S6.3. The HRMS data for $[M+3H]^{3+}$ ion of [$C_{60}H_{102}N_{22}O_{12}$] (compound **2**).

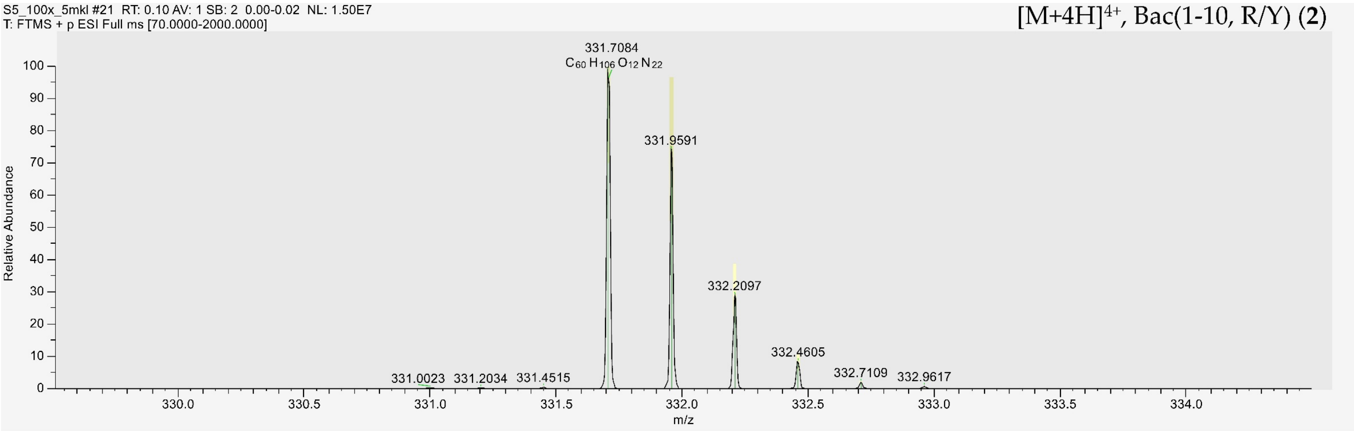

Peak Mass	Display Formula	Delta [ppm]	Theo. mass	Pattern Cov. [%]
331.7084	$C_{60}H_{106}O_{12}N_{22}$	-0.15	331.7085	99.85

Figure S6.4. The HRMS data for $[M+4H]^{4+}$ ion of [$C_{60}H_{102}N_{22}O_{12}$] (compound **2**).

HRMS data for Bac(1–10, R/Y)-C2-TPP (3)

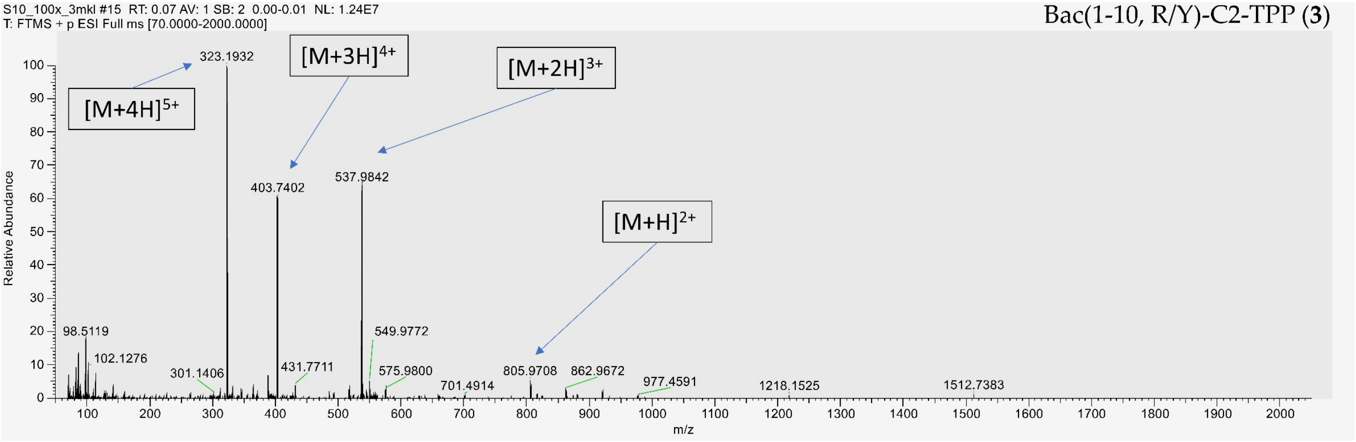

Figure S7.1. The HRMS spectrum of compound **3** in positive ion mode.

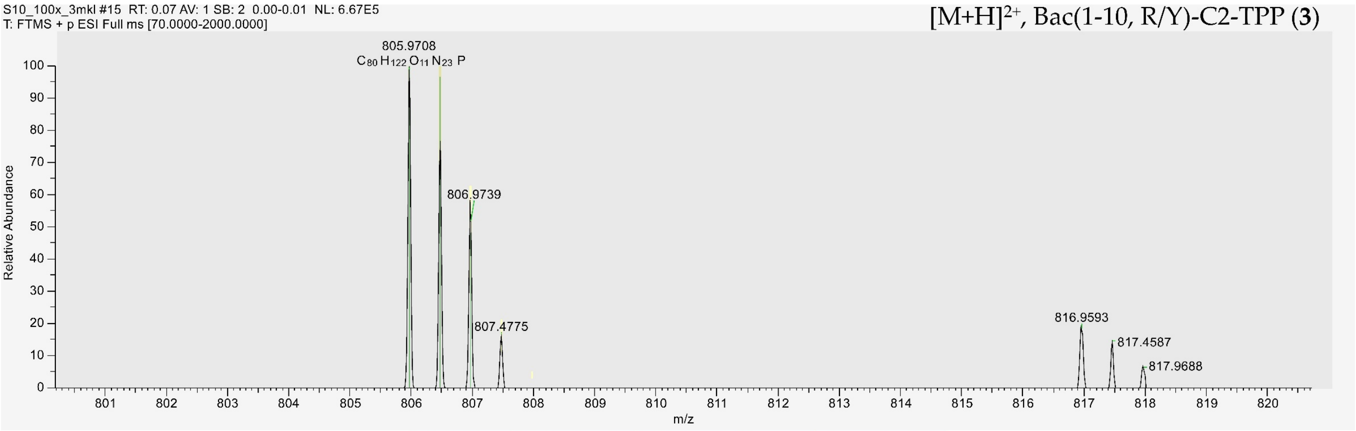

Peak Mass	Display Formula	Delta [ppm]	Theo. mass	Pattern Cov. [%]
805.9708	$C_{80}H_{122}O_{11}N_{23}P$	-0.3	805.971	98.48

Figure S7.2. The HRMS data for $[M+H]^{2+}$ ion of $[C_{80}H_{121}N_{23}O_{11}P]^{+}$ (compound **3**).

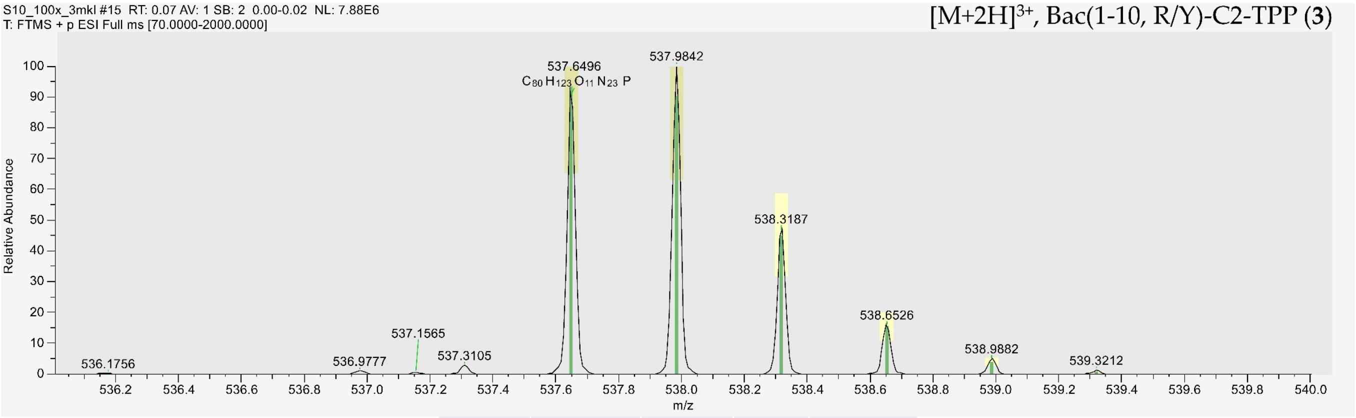

Peak Mass	Display Formula	Delta [ppm]	Theo. mass	Pattern Cov. [%]
537.6496	$C_{80}H_{123}O_{11}N_{23}P$	-0.32	537.6498	99.95

Figure S7.3. The HRMS data for $[M+2H]^{3+}$ ion of $[C_{80}H_{121}N_{23}O_{11}P]^{+}$ (compound **3**).

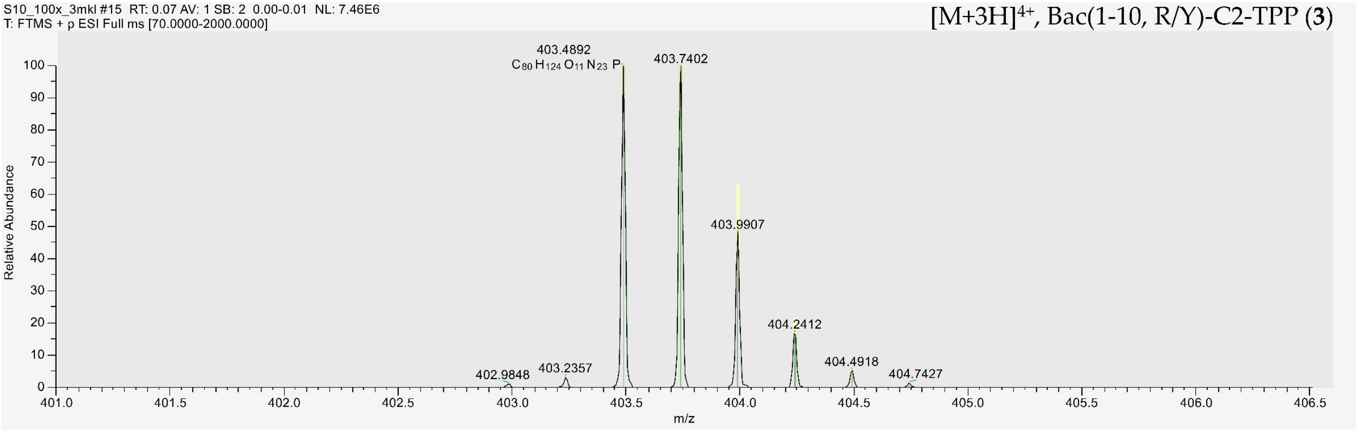

Peak Mass	Display Formula	Delta [ppm]	Theo. mass	Pattern Cov. [%]
403.4892	$C_{80}H_{124}O_{11}N_{23}P$	0.16	403.4892	99.95

Figure S7.4. The HRMS data for $[M+3H]^{4+}$ ion of $[C_{80}H_{121}N_{23}O_{11}P]^+$ (compound **3**).

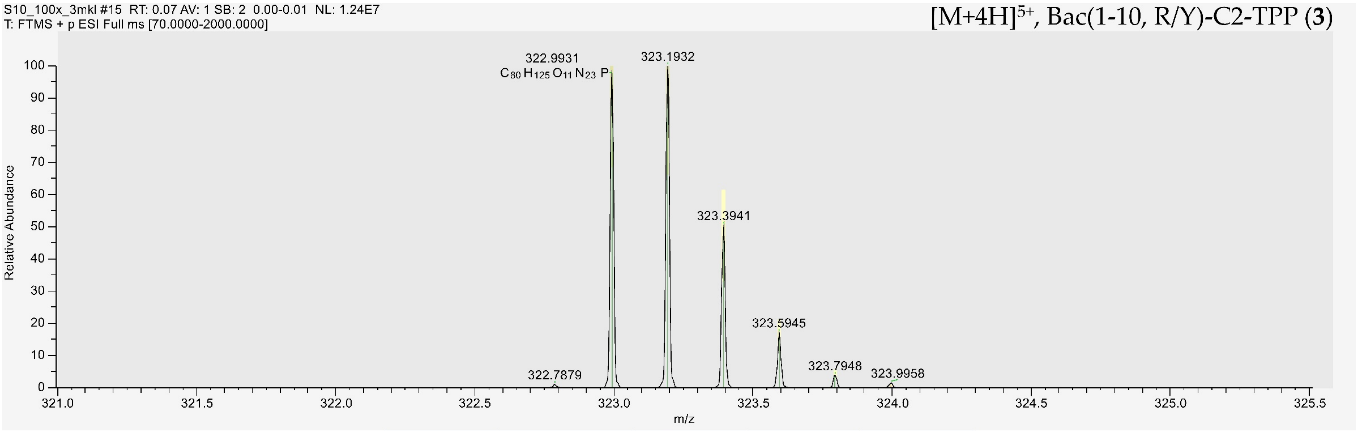

Peak Mass	Display Formula	Delta [ppm]	Theo. mass	Pattern Cov. [%]
322.9931	$C_{80}H_{125}O_{11}N_{23}P$	0.89	322.9928	99.59

Figure S7.5. The HRMS data for $[M+4H]^{5+}$ ion of $[C_{80}H_{121}N_{23}O_{11}P]^+$ (compound **3**).

HRMS data for Bac(1–10, R/Y)-C10-TPP (4)

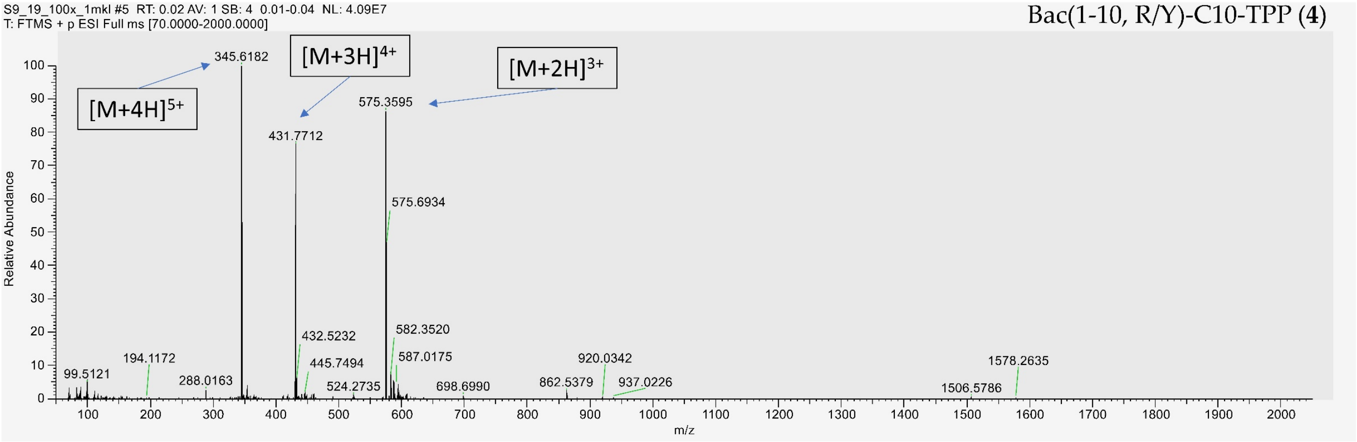

Figure S8.1. The HRMS spectrum of compound **4** in positive ion mode.

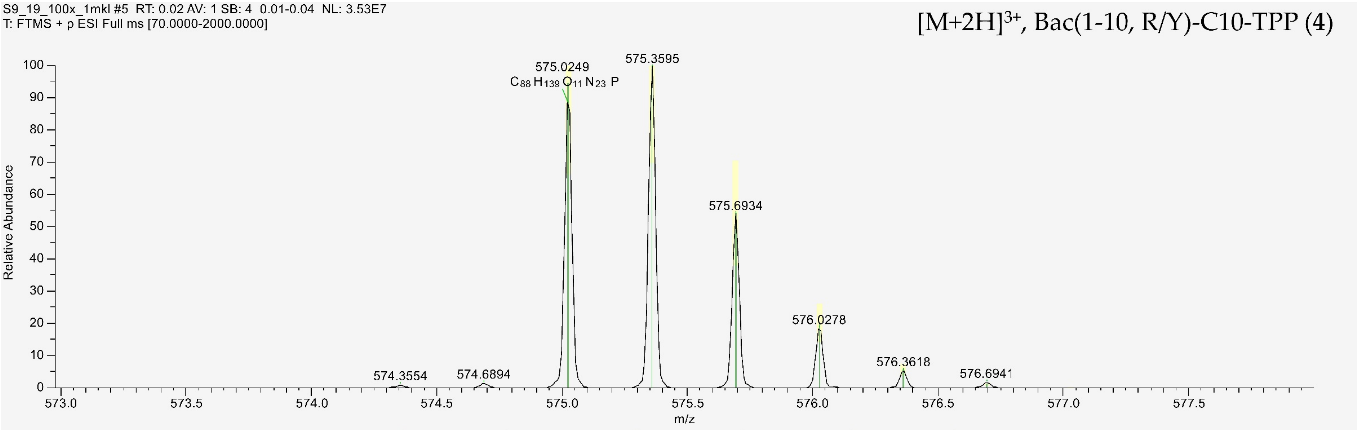

Peak Mass	Display Formula	Delta [ppm]	Theo. mass	Pattern Cov. [%]
575.0249	$C_{88}H_{139}O_{11}N_{23}P$	0.02	575.0249	99.92

Figure S8.2. The HRMS data for $[M+2H]^{3+}$ ion of $[C_{88}H_{137}N_{23}O_{11}P]^{+}$ (compound **4**).

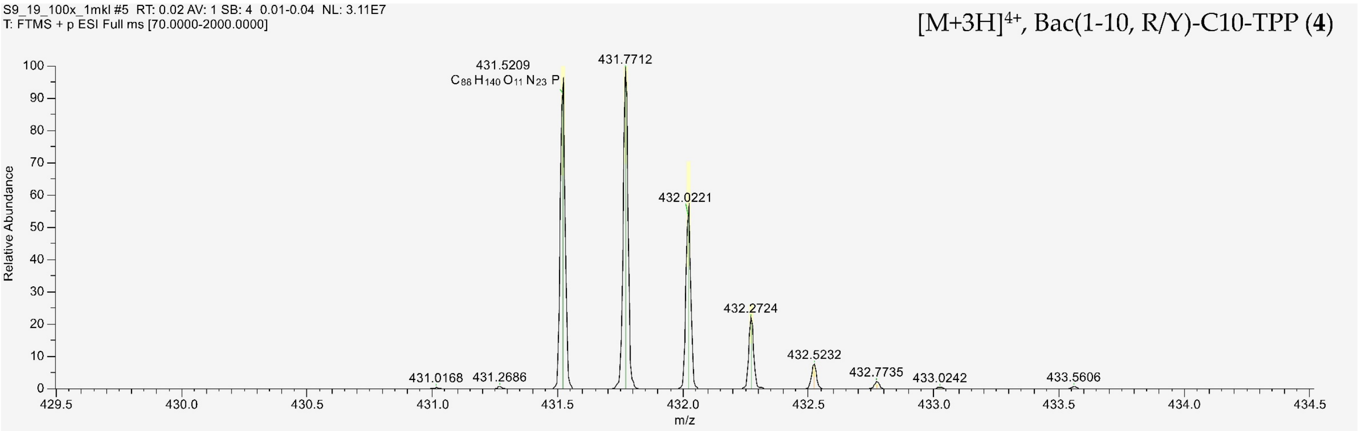

Peak Mass	Display Formula	Delta [ppm]	Theo. mass	Pattern Cov. [%]
431.5209	$C_{88}H_{140}O_{11}N_{23}P$	0.93	431.5205	97.35

Figure S8.3. The HRMS data for $[M+3H]^{4+}$ ion of $[C_{88}H_{137}N_{23}O_{11}P]^{+}$ (compound **4**).

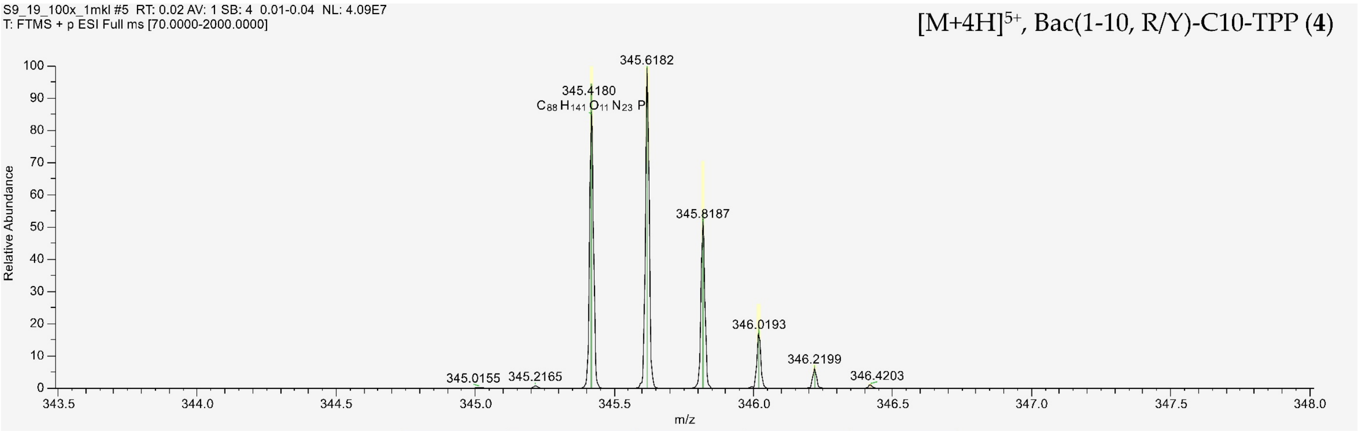

Peak Mass	Display Formula	Delta [ppm]	Theo. mass	Pattern Cov. [%]
345.418	$C_{88}H_{141}O_{11}N_{23}P$	0.44	345.4178	99.44

Figure S8.4. The HRMS data for $[M+4H]^{5+}$ ion of $[C_{88}H_{137}N_{23}O_{11}P]^{+}$ (compound **4**).

HRMS data for TPP-C10-Bac(1–10, R/Y) (5)

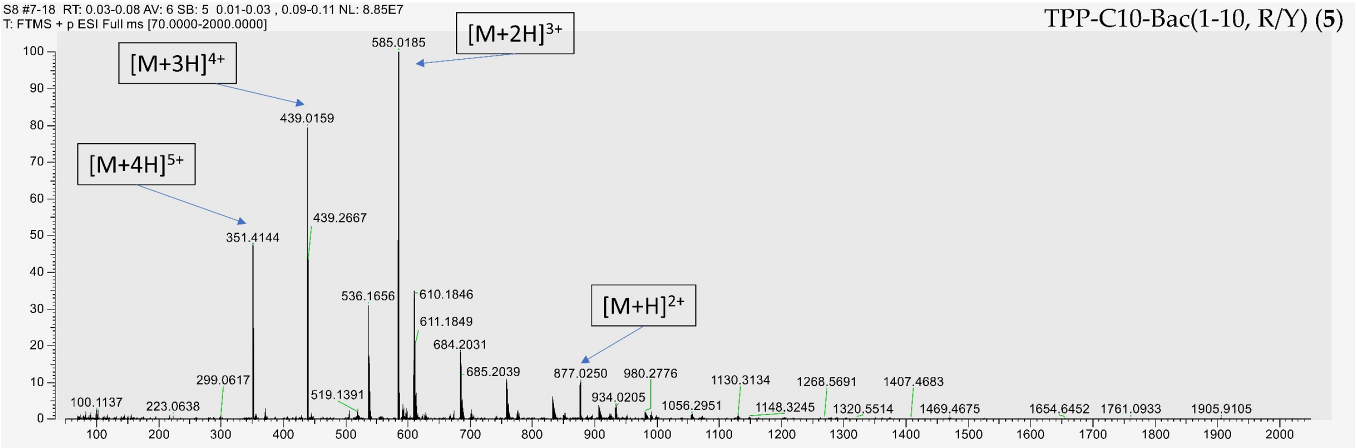

Figure S9.1. The HRMS spectrum of compound **5** in positive ion mode.

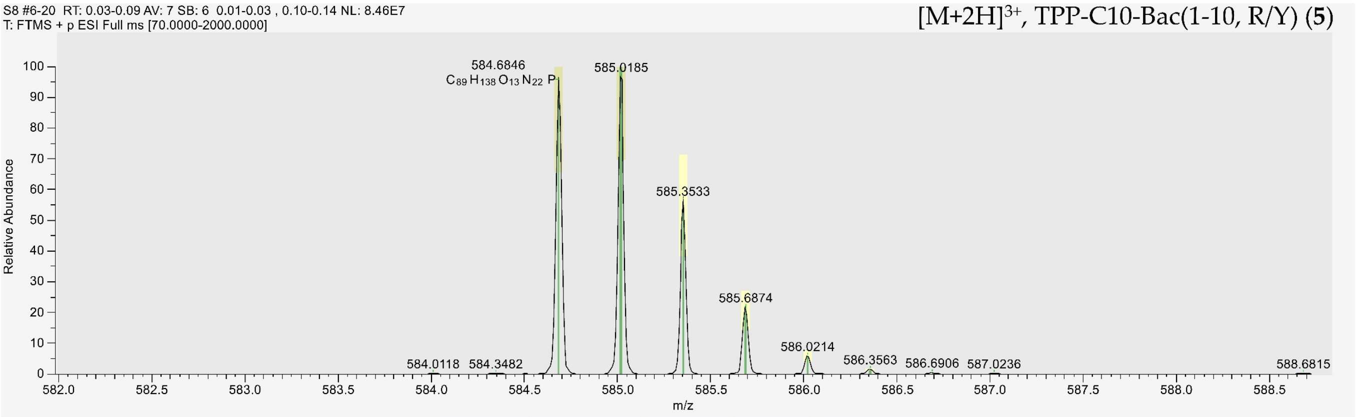

Peak Mass	Display Formula	Delta [ppm]	Theo. mass	Pattern Cov. [%]
584.6846	$C_{89}H_{138}O_{13}N_{22}P$	0.11	584.6845	100

Figure S9.2. The HRMS data for $[M+2H]^{3+}$ ion of $[C_{89}H_{136}N_{22}O_{13}P]^{+}$ (compound **5**).

IV. HPLC DATA

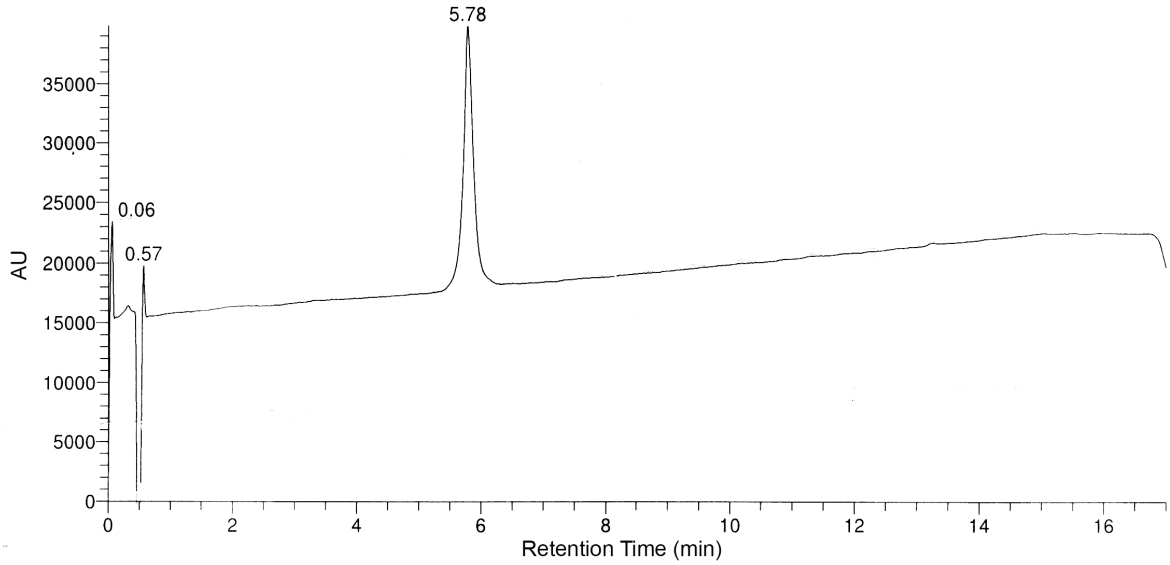

Figure S10.1. HPLC analysis of Onc112 (retention time: 5.78 min) in a gradient of CH_3CN in 0.05% TFA from 5 to 55% for 17 min.

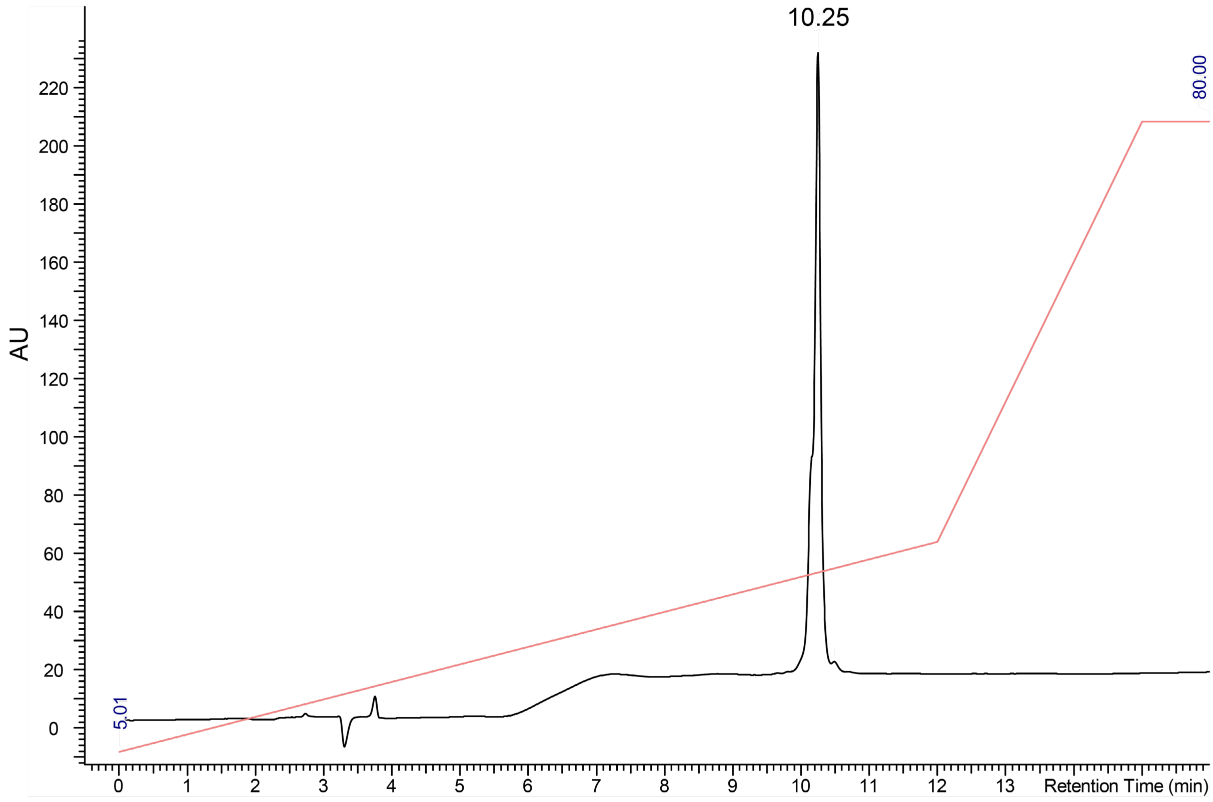

Figure S10.2. HPLC analysis of compound **1**, Bac(1–10), (retention time: 10.25 min) in a gradient of CH_3CN in 0.05% TFA from 5 to 30% for 12 min and from 30 to 80% for 3 min.

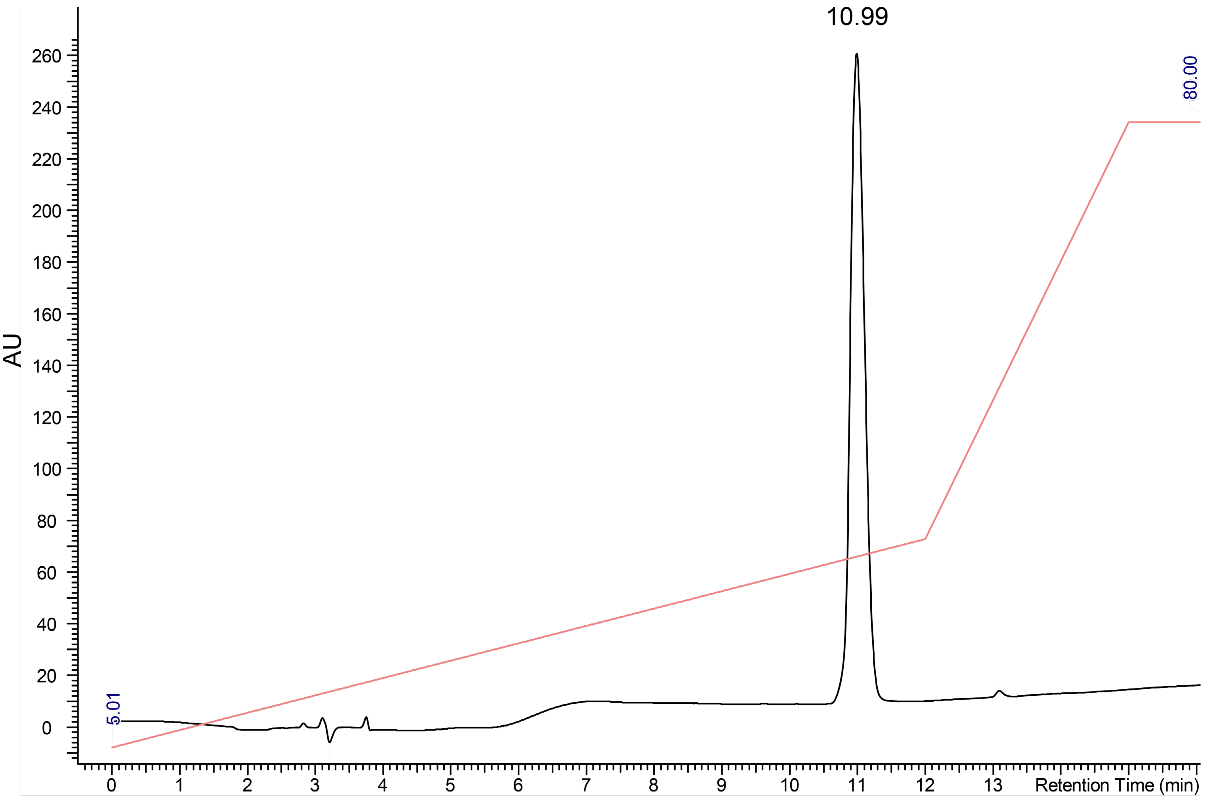

Figure S10.3. HPLC analysis of compound **2**, Bac(1–10, R/Y), (retention time: 10.99 min) in a gradient of CH_3CN in 0.05% TFA from 5 to 30% for 12 min and from 30 to 80% for 3 min.

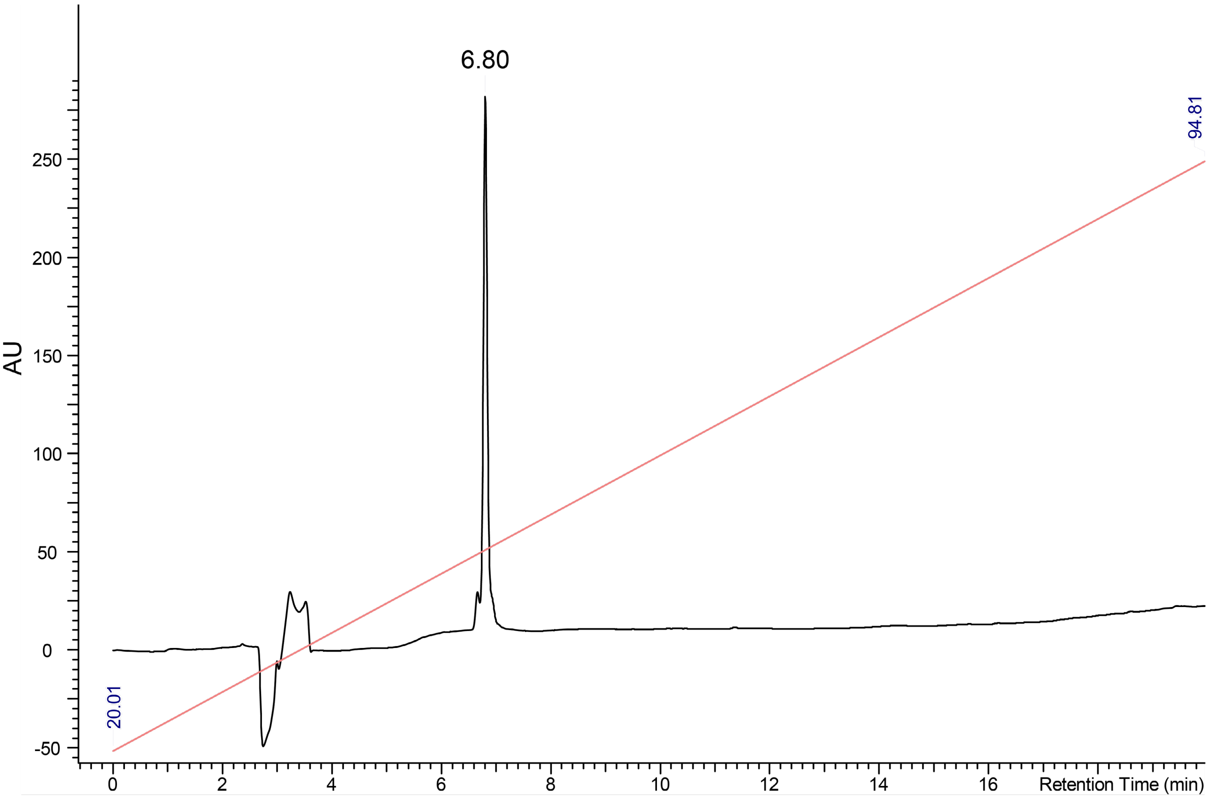

Figure S10.4. HPLC analysis of compound **3**, Bac(1–10, R/Y)-C2-TPP, (retention time: 6.80 min) in a gradient of CH_3CN in 0.05% TFA from 20 to 95% for 20 min.

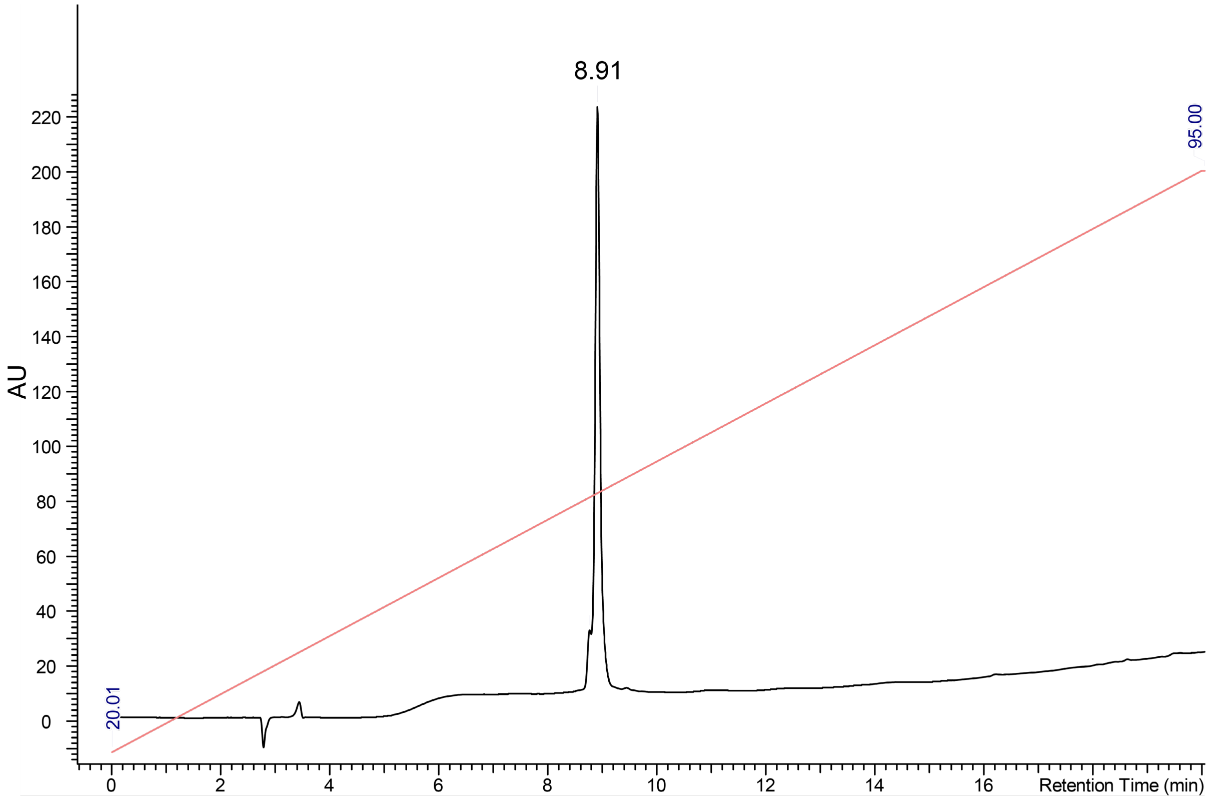

Figure S10.5. HPLC analysis of compound **4**, Bac(1–10, R/Y)-C10-TPP, (retention time: 8.91 min) in a gradient of CH_3CN in 0.05% TFA from 20 to 95% for 20 min.

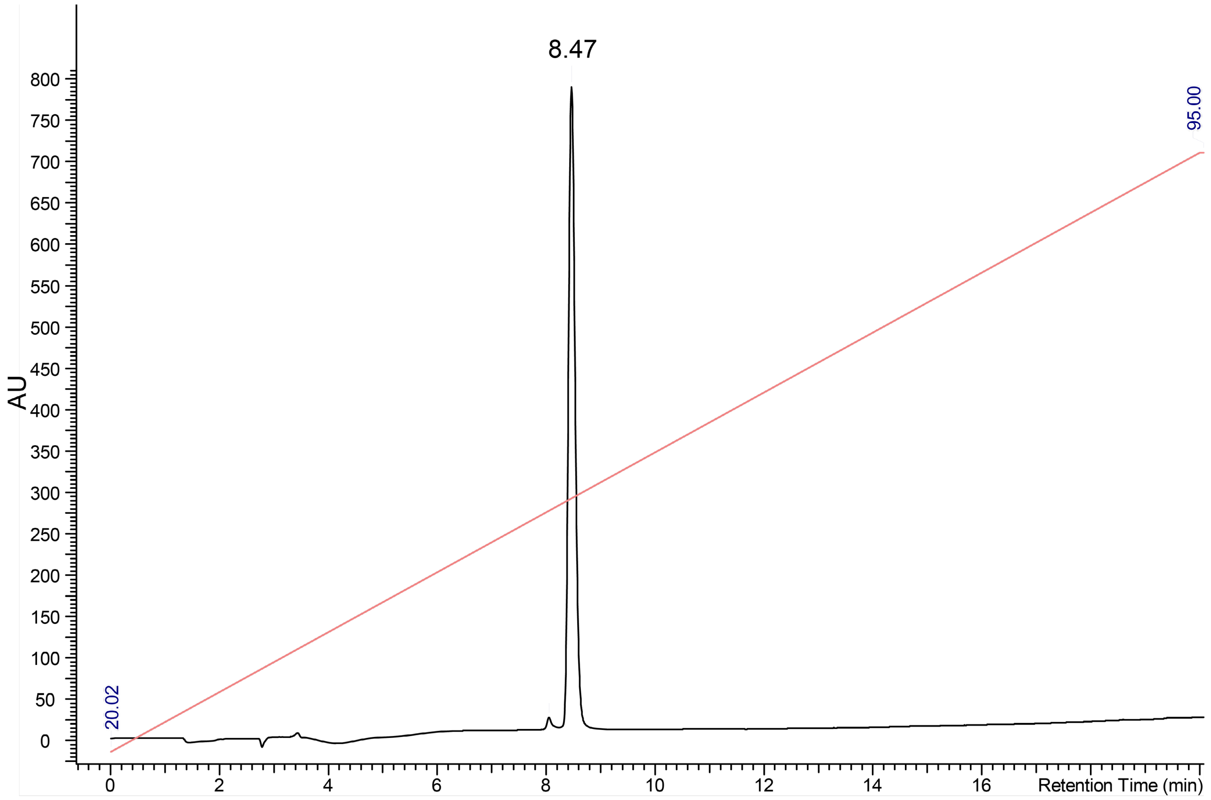

Figure S10.6. HPLC analysis of compound **5**, TPP-C10-Bac(1–10, R/Y), (retention time: 8.47 min) in a gradient of CH_3CN in 0.05% TFA from 20 to 95% for 20 min.

V. MTT-ASSAY DATA

Two thousand five hundred cells per well for the MCF7, HEK293T, and A549 cell lines or 4000 cells per well for the VA13 cell line were plated in 135 μL of DMEM-F12 media with 10% FBS (both Gibco, Waltham, MA, USA) in a 96-well plate and were incubated in a 5% CO_2 incubator for the first 16 h, without treatment. Then 15 μL of media-DMSO solutions of the tested substances were added to the cells (the final DMSO concentrations in the media were 1% or less), and the cells were treated for 72 h with 25 nM–50 μM (eight dilutions) of the tested substances (triplicate each). Serial dilutions of DMSO and doxorubicin were used as controls. The MTT reagent (Paneco LLC, Moscow, Russia) was then added to the cells at a final concentration of 0.5 g/L (10× stock solution in PBS was used) and incubated for 2.5 h at 37 °C in the incubator under an atmosphere of 5% CO_2. The MTT solution was then discarded, and 140 μL of DMSO (PharmaMed LLC, Krasnodar, Russia) was added. The plates were swayed on a shaker (60 rpm) to dissolve the formazan. The absorbance was measured using the VICTOR X5 Multilabel Plate Reader (PerkinElmer, Waltham, MA, USA) at a wavelength of 565 nm (in order to measure formazan concentration). The results were used to construct a dose-response graph and to estimate IC_{50} values.

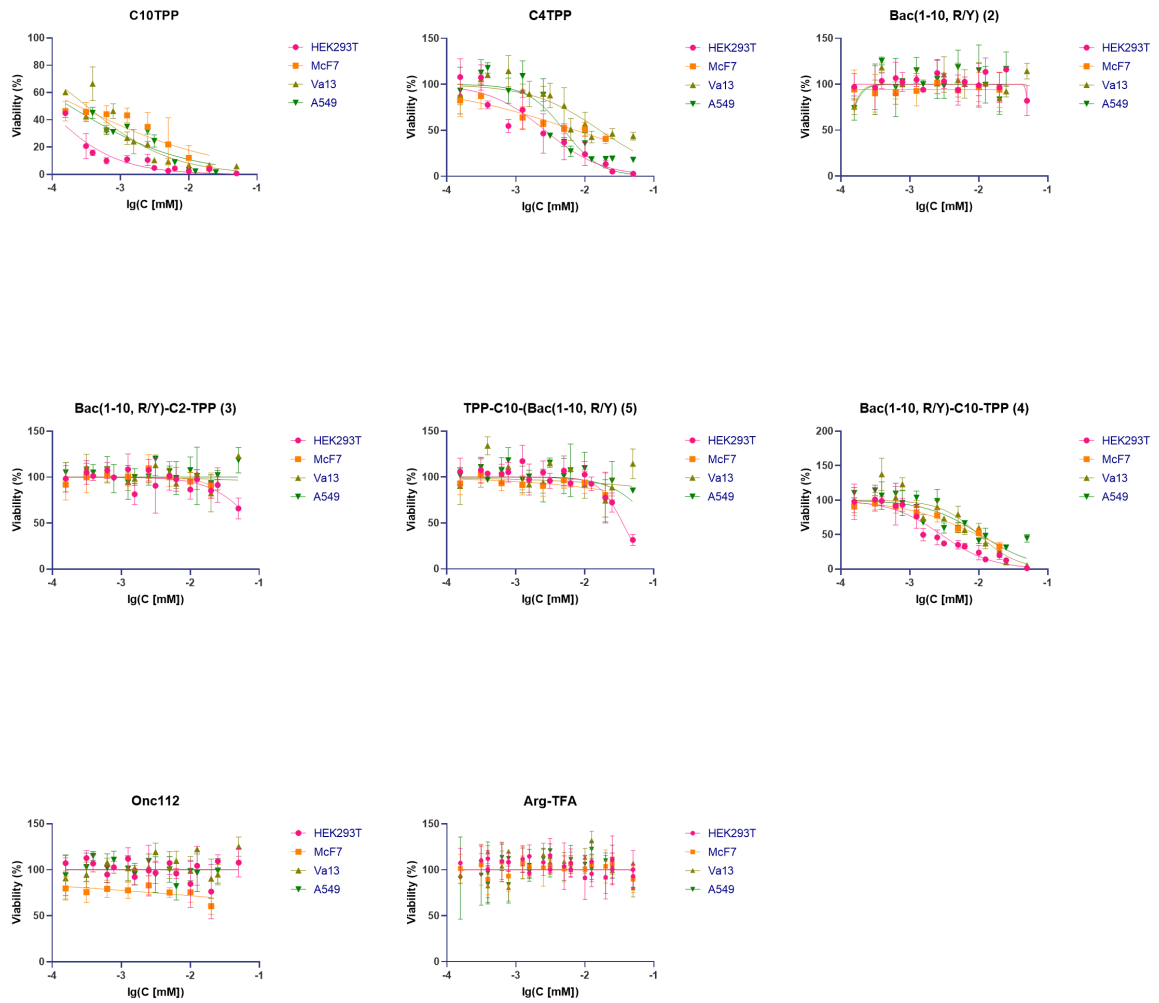

Figure S11. The dependencies of viability of cells after treatment by tested compounds.

VI. SUPPLEMENTARY REFERENCES

1. Hansen, P. R.; Oddo, A. Fmoc solid–phase peptide synthesis. *Methods Mol. Biol.* **2015**, *1348*, 33-50, doi: 10.1007/978-1-4939-2999-3_5.

2. Eissler, S.; Kley, M.; Bächle, D.; Loidl, G.; Meier, T.; Samson, D. Substitution determination of Fmoc-substituted resins at different wavelengths. *J. Pept. Sci.* **2017**, *23*, 757-762, doi: 10.1002/psc.3021.

3. Kaiser, E.; Colescott, R. L.; Bossinger, C. D.; Cook, P. I. Color test for detection of free terminal amino groups in the solidphase synthesis of peptides. *Anal. Biochem.*, **1970**, *34*, 595-598, doi: 10.1016/0003-2697(70)90146-6.
